# Matricellular protein SMOC2 safeguards tubular integrity in acute kidney injury via integrin β3-dependent inhibition of CCND1-CDK4/6 axis

**DOI:** 10.1186/s43556-026-00407-6

**Published:** 2026-02-10

**Authors:** Peng Gao, Schrodinger Cenatus, Dan Zhang, Siwei Chu, Nathalie Henley, Vincent Pichette, Jonatan Barrera-Chimal, Casimiro Gerarduzzi

**Affiliations:** 1 Department of Pharmacology and Physiology, Faculty of Medicine, University of Montreal, Montreal, QC Canada; 2 Maisonneuve-Rosemont Hospital Research Center, Center affiliated with the University of Montreal, Montreal, QC Canada; 3 Department of Biochemistry and Molecular Medicine, Faculty of Medicine, University of Montreal, Montreal, QC Canada; 4 Department of Nephrology, Affiliated Hospital of Xuzhou Medical University, Xuzhou, China; 5 Department of Nephrology, Maisonneuve-Rosemont Hospital, Montreal, QC Canada

**Keywords:** SMOC2, Acute tubular injury, CCND1, Cell cycle arrest, DNA damage, Renal fibrosis

## Abstract

Tubular injury during acute kidney injury (AKI) is a major determinant of chronic kidney disease (CKD) progression, yet the molecular mechanisms underlying tubular protection remain incompletely understood. Here, we identify the matricellular protein SPARC-related modular calcium-binding 2 (SMOC2) as a previously unrecognized protective regulator of tubular injury. Although SMOC2 has been implicated in renal fibrosis through fibroblast activation, its role during AKI remains unknown. We show that SMOC2 expression is rapidly and robustly induced in renal tubules following exposure to aristolochic acid I (AAI) or cisplatin. Unexpectedly, SMOC2 knockout mice exhibited aggravated tubular injury, increased DNA damage and apoptosis, and worsened renal function in both AAI- and cisplatin-induced AKI models, whereas recombinant SMOC2 (rSMOC2) treatment markedly ameliorated AAI-induced tubular injury. Furthermore, in an AAI-induced AKI-to-CKD model, SMOC2 deficiency exacerbated renal fibrosis, linking early tubular protection to long-term outcomes. Mechanistically, transcriptomic profiling and biochemical analyses revealed that SMOC2 suppresses aberrant G1/S cell cycle progression by restraining the CCND1-CDK4/6 axis through its interaction with integrin β3 (ITGB3), thereby arresting tubular cells in the G1 phase and facilitating DNA repair. This interaction depends on the cooperation of multiple structural domains rather than a single motif. Notably, pharmacological inhibition of CDK4/6 with palbociclib phenocopied the protective effects of SMOC2, with post-injury treatment providing superior protection, thus defining a druggable downstream pathway. Collectively, our findings uncover a previously unappreciated cytoprotective role of SMOC2 in AKI and establish the SMOC2-ITGB3-CCND1-CDK4/6 signaling axis as a potential therapeutic target to prevent AKI progression and its transition to CKD.

**Supplementary Information:**

The online version contains supplementary material available at 10.1186/s43556-026-00407-6.

## Introduction

Acute kidney injury (AKI) is defined as a sudden decline in kidney function, typically marked by a rapid increase in serum creatinine (Scr), a decrease in urine output, or both [1]. It is increasingly recognized as a major risk factor for the development of chronic kidney disease (CKD) [2–5]. AKI is a complex pathological process involving tubular epithelial cells (TECs), endothelial cells, and infiltrating inflammatory cells [6, 7]. Among these, injury to TECs plays a central role in disease progression because TECs are directly exposed to toxic filtrate, highly metabolically active, and essential for maintaining renal structure and function; accordingly, tubular injury is a key driver of AKI-to-CKD transition [8, 9].

In response to mild injury, an adaptive repair process is initiated, involving dedifferentiation and proliferation of surviving TECs to restore tubular integrity [10]. However, when AKI is severe or recurrent, this repair process frequently becomes maladaptive, leading to chronic inflammation and renal interstitial fibrosis, thereby accelerating the transition from AKI to CKD [11, 12]. Notably, injured TECs can adopt a secretory phenotype, releasing factors such as cytokines, chemokines, and extracellular matrix (ECM)-modulating molecules that act in autocrine and paracrine manners on tubules and neighboring cells (e.g., fibroblasts), thereby influencing tubular repair or promoting fibrosis [11, 13]. Among ECM-modulating molecules, matricellular proteins (MCPs) have emerged as critical regulators of tubular repair and fibrosis [14, 15]. Unlike structural ECM proteins, which primarily provide mechanical support, MCPs are matrix constituents that function as dynamic modulators of the cellular microenvironment. By interacting with cellular surface receptors, MCPs can influence key cellular processes such as adhesion, proliferation, migration, and survival in a context-dependent manner [14, 15]. Thus, a deeper understanding of the matrisome of injured TECs is essential for unraveling the mechanisms that govern tubular repair and fibrosis.

Building on this concept, our previous studies have characterized expression profiles of MCPs in two renal fibrotic models: folic acid-induced and unilateral ureteral obstruction (UUO)-induced fibrosis [16]. Among the differentially expressed MCPs, SPARC-related modular calcium-binding protein 2 (SMOC2) emerged as one of the most highly upregulated MCPs in both acute and chronic kidney injury, while exhibiting low expression in healthy kidneys [16–18]. SMOC2 is a member of the SPARC (secreted protein acidic and rich in cysteine) family of MCPs and structurally comprises a follistatin-like (FS) domain, two thyroglobulin (TG) domains, an extracellular calcium-binding (EC) domain, and a unique SMOC domain [19]. Through interactions with cell-surface receptors such as integrins, SMOC2 exerts diverse biological functions and has been implicated in tumor development and metastasis, by modulating angiogenesis [20], cellular proliferation [21–24], migration [25], and epithelial-to-mesenchymal transition (EMT) [26]. Beyond its role in cancer, SMOC2 has also been linked to fibrosis in multiple organs, including the liver, lungs, and heart, by potentiating the TGF-β signaling cascade [27, 28] or mitogen-activated protein kinase (MAPK) pathway [29]. Our lab, along with independent studies, has demonstrated that SMOC2 promotes fibroblast activation in chronic renal fibrosis models by interacting with integrins β1 and 5 [17, 30]. However, its function in early tubular injury and repair during AKI remains poorly understood. Given its early upregulation in renal tubules after kidney injury, we hypothesize that SMOC2 actively modulates tubular injury responses before influencing fibroblast activation and fibrosis development.

To investigate SMOC2’s role in tubular injury and repair, we utilized SMOC2 knockout (KO) mice in two complementary acute tubular injury models: aristolochic acid I (AAI)-induced nephropathy (AAN) and cisplatin-induced AKI. AAI selectively enters TECs via organic anion transporters (OATs) 1–3 [31, 32], while cisplatin preferentially accumulates in TECs through organic cation transporter 2 (OCT2) [33, 34]. Both agents induce the formation of DNA crosslinks and/or adducts, triggering the DNA damage response that leads to specific tubular injury [35–37]. By leveraging these models, we aim to elucidate how SMOC2 is implicated in the tubular response to AKI, as well as its role in AKI-to-CKD progression.

## Results

### SMOC2 is broadly induced in renal tubules in AAN kidneys

**Fig. 1:**
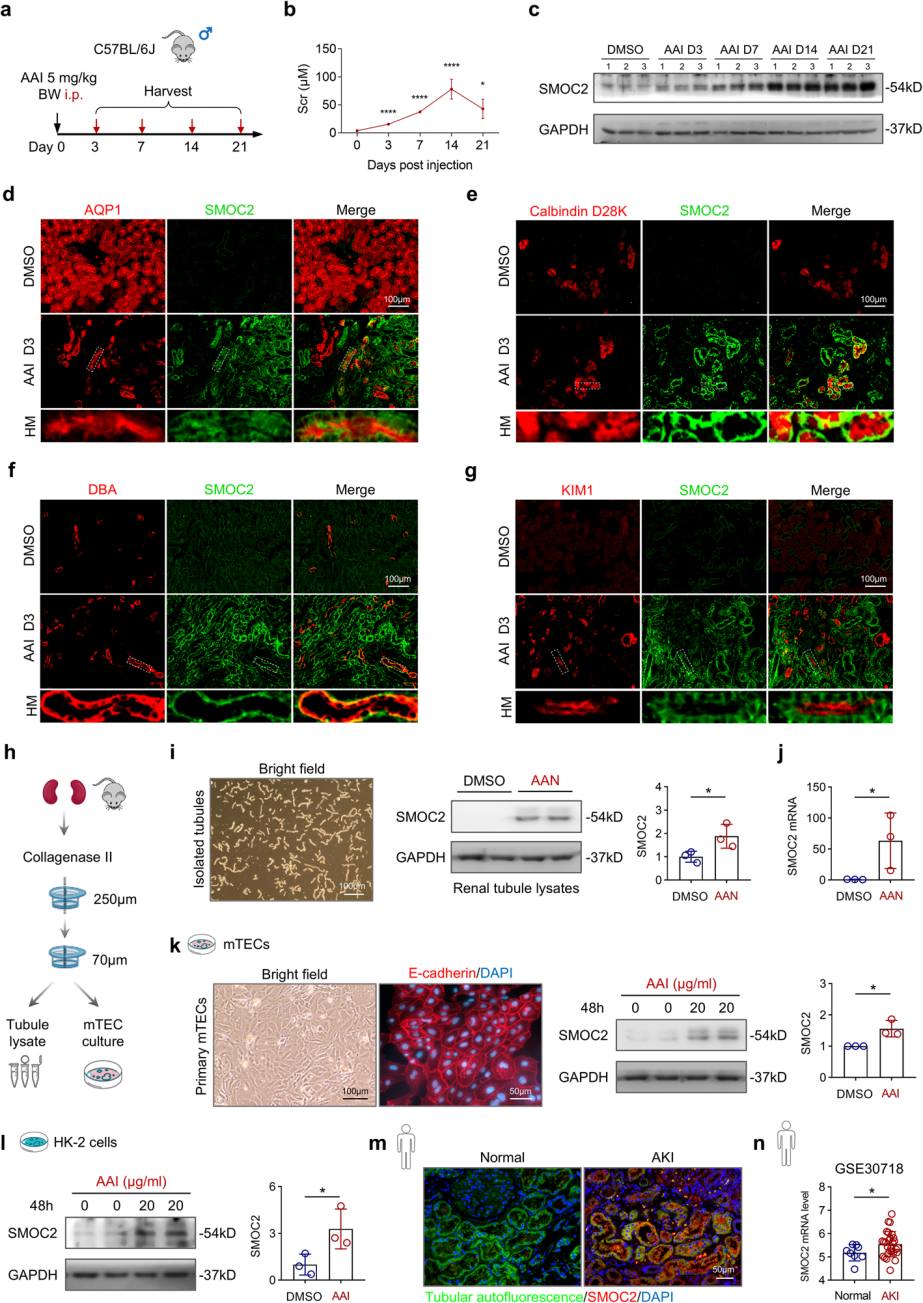
Aristolochic acid I (AAI) toxicity leads to renal dysfunction and increased SMOC2 expression in renal tubular epithelial cells. **a** Schematic representation of the experimental design for sample collection in the aristolochic acid nephropathy (AAN) model. Male C57BL/6 J mice were administered a single intraperitoneal (i.p.) injection of 5 mg/kg body weight (BW) AAI or vehicle (DMSO) and sacrificed at days 3, 7, 14, and 21 (*n* = 6 per time point). **b** Time-course changes in serum creatinine (Scr) levels following AAI injection. *p < 0.05, ****p < 0.0001 vs. DMSO group. **c** Western blot analysis of SMOC2 expression in kidney tissues from AAN mice. **d**-**g** Co-immunostaining of SMOC2 with specific tubular markers: proximal tubules (AQP1), distal tubules (Calbindin D28K), collecting ducts (DBA), and injured tubules (KIM1). **h** Schematic illustration of the renal tubule isolation procedure and the generation of primary mouse tubular epithelial cells (mTECs) cultures. **i**, **j** Representative images of freshly isolated tubules from mouse kidneys, which were subsequently analyzed by Western blot and qPCR to assess SMOC2 expression in tubules isolated from AAI and DMSO-treated mice. **k** Representative images of primary mTECs derived from isolated renal tubules, shown under bright field and validated for epithelial purity by E-cadherin staining. Western blot analysis and quantification of SMOC2 expression in mTECs treated with AAI or DMSO. **l** Western blot and quantification analysis of SMOC2 expression in AAI- and DMSO-treated human kidney tubular epithelial cells (HK-2). **m** Representative immunofluorescence images of SMOC2 expression in human kidney biopsy samples from patients with AKI (*n* = 3) and in normal kidney tissues adjacent to renal carcinoma (*n* = 2) used as controls. Green indicates tubular epithelial cell autofluorescence. **n** The mRNA level of SMOC2 in the kidney from both AKI patients and healthy controls (RNA-seq data were derived from GSE30718). *n* = 3 for in vitro, *p < 0.05, **p < 0.01, ***p < 0.001, ****p < 0.0001

To induce acute tubular injury, male C57BL/6 J mice received a single injection of aristolochic acid I (AAI; 5 mg/kg body weight, BW) [38], and serum and kidney samples were collected on days 3, 7, 14, and 21 (Fig. 1a). Renal function analysis revealed that Scr levels progressively increased, peaking at day 14 before declining by day 21 (Fig. 1b). Hematoxylin and eosin (HE) staining together with semi-quantitative injury scoring revealed the sequential histopathological changes during AKI progression (Fig. S1a, b). By day 3, TECs exhibited marked swelling and cytoplasmic vacuolar degeneration, accompanied by narrowing of the interstitial space. By day 7, the kidney showed extensive tubular damage characterized by prominent luminal accumulation of necrotic debris (asterisk), along with the formation of proteinaceous casts (arrowhead). By days 14–21, tubular atrophy (#) and pronounced interstitial inflammatory infiltration were observed, indicating a transition from AKI to CKD.

Previous studies have reported that SMOC2 is expressed in renal tubules and interstitial fibroblasts in CKD patients and in mice with UUO [17]. However, its pathological expression pattern in AKI remains poorly defined. Western blot analysis revealed a time-dependent increase in SMOC2 expression following AAI injection compared with control kidneys (Fig. 1c and Fig. S1c). Co-immunostaining of SMOC2 with tubular segment-specific markers revealed that SMOC2 expression was broadly upregulated during the acute phase (day 3) following AAI injection, predominantly within the tubular compartment, as demonstrated by its co-localization with AQP1⁺ proximal tubules (Fig. 1d), Calbindin D28K⁺ distal tubules (Fig. 1e), DBA⁺ collecting ducts (Fig. 1f), and KIM1⁺ injured tubules (Fig. 1g).

To further validate its tubular expression pattern, renal tubules were isolated from DMSO- and AAI-injected mice using a collagenase II-based digestion method (Fig. 1h). Western blot and qPCR analyses confirmed a significant increase in SMOC2 protein (Fig. 1i) and mRNA (Fig. 1j) levels in tubules isolated from AAN kidneys, corroborating its tubular induction in vivo. In vitro, SMOC2 expression was markedly upregulated in both primary mouse TECs (mTECs; Fig. 1k) and human proximal TECs (HK-2; Fig. 1l) following treatment with 20 μg/mL AAI for 48 h. To assess the clinical relevance of SMOC2 in AKI, we next examined its expression in human AKI kidney tissues. Immunofluorescence staining revealed minimal SMOC2 expression in normal kidneys, whereas robust upregulation was observed in AKI kidneys, predominantly within renal tubules (Fig. 1m). Consistently, bulk RNA-sequencing analysis of an independent human AKI cohort confirmed a significant increase in renal SMOC2 transcript levels compared with controls (Fig. 1n). Collectively, these findings demonstrate that SMOC2 is robustly induced in TECs following AKI across experimental models and human disease.

### SMOC2 deficiency exacerbates AAI-induced acute tubular injury in male mice

**Fig. 2:**
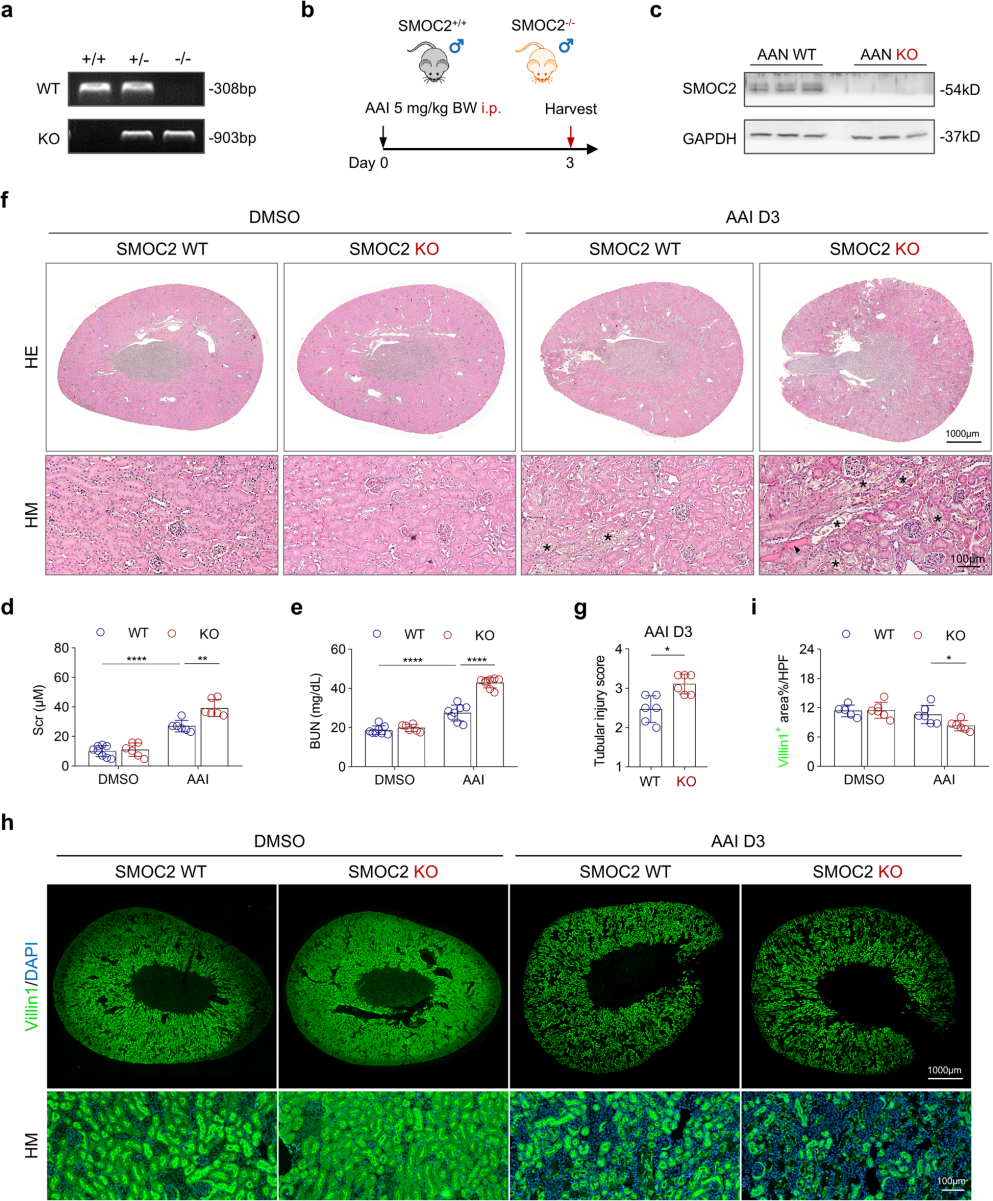
SMOC2 knockout exacerbates acute tubular injury in male AAN mice. **a** Genotyping results confirming SMOC2 WT and KO mice. **b** Schematic diagram illustrating the AAI-induced acute tubular injury model. **c** Western blot analysis of SMOC2 expression in kidney tissues from AAN WT and KO mice. **d, e** Scr and BUN levels in DMSO- or AAI-injected mice at day 3. **f** Upper panel: Representative HE-stained transverse kidney sections from mice treated with DMSO or AAI. Lower panel: Higher magnification (HM) images highlighting tubular injury in each group. **g** Tubular injury score in WT and KO AAN mice at day 3. **h** Upper panel: Representative images of Villin1 staining, a mature TECs marker, in transverse kidney sections from DMSO- or AAI-treated mice. Lower panel: HM images highlighting the loss of Villin1 expression in AAI-treated mice. **i** Quantification of Villin1.^+^ area per high-power field (× 400, HPF) in DMSO- or AAI-injected WT and KO mice at day 3. *n* = 6 for each group, *p < 0.05, **p < 0.01, ***p < 0.001, ****p < 0.0001

To assess the role of SMOC2 in tubular injury in vivo, global SMOC2 KO mice were generated and validated at the genomic level (Fig. 2a). SMOC2 wild type (WT) and KO mice were then administered a single injection of AAI (5 mg/kg BW) and euthanized on day 3 (Fig. 2b). Western blot analysis confirmed efficient deletion of SMOC2 protein in the kidneys of SMOC2 KO AAN mice (Fig. 2c). Under basal conditions, SMOC2 WT and KO mice showed no significant differences in Scr (Fig. 2d), blood urea nitrogen (BUN; Fig. 2e), or renal histology at 2–3 months of age (Fig. 2f). HE staining revealed that AAI-induced tubular damage was markedly exacerbated in SMOC2 KO mice (Fig. 2f, g). Consistently, Villin 1 staining, a marker of differentiated renal tubular epithelium [39], demonstrated significantly greater brush border loss in SMOC2 KO mice (Fig. 2h, i). Given the known sex-based differences in AKI susceptibility, we also examined the effects of AAI in female mice. Even when higher AAI doses (Fig. S2) or repeated injection protocols (Fig. S3) were applied, female mice did not develop overt tubular injury, and no genotype-dependent differences were observed (Fig. S3). Consistent with previous reports [40], female mice are resistant to AAI-induced kidney injury. Importantly, under these dosing conditions, no detectable hepatic injury was observed (Fig. S3), confirming that the AAI regimens used preferentially induced renal, rather than hepatic, toxicity. Therefore, all subsequent experiments were performed in male mice to more accurately evaluate the impact of SMOC2 deficiency on tubular injury. Collectively, these results demonstrate that SMOC2 deficiency aggravates AAI-induced acute tubular injury in male mice, supporting a protective role for SMOC2 during AKI.

### SMOC2 deficiency exacerbates AAI-induced DNA damage

**Fig. 3:**
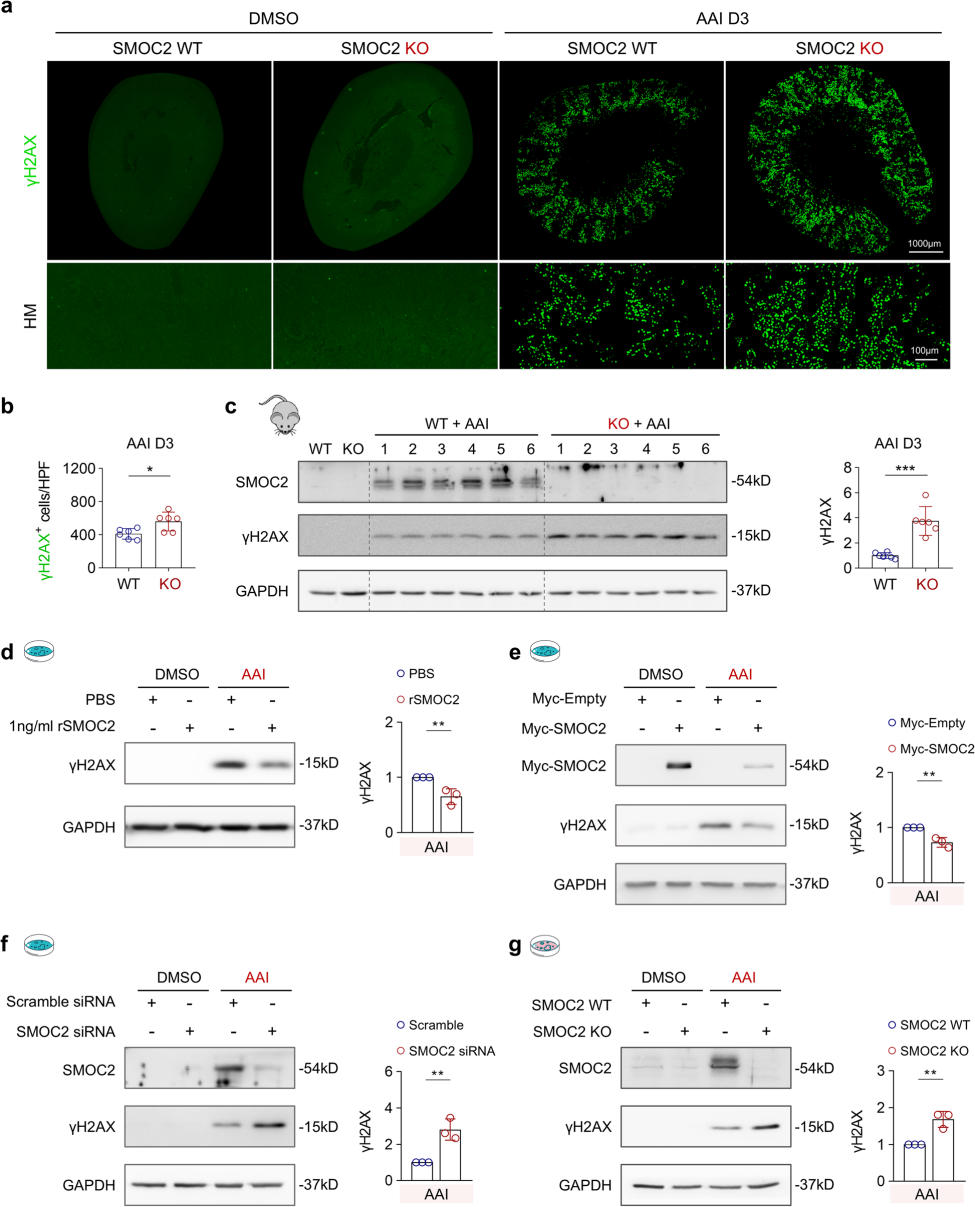
SMOC2 knockout exacerbates DNA damage in male AAN mice and AAI-treated renal tubular cells. **a** Upper panel: Representative images of γH2AX staining, a DNA damage marker, in transverse kidney sections from DMSO- or AAI-injected mice. Lower panel: HM images highlighting γH2AX^+^ cells in each group. **b** Quantification of γH2AX.^+^ cells per HPF in AAI-injected SMOC2 WT and KO mice at day 3. **c** Western blot and quantitative analysis of γH2AX expression in kidney tissues from WT and SMOC2 KO mice on day 3 following DMSO or AAI injection. **d** Western blot and quantification of γH2AX expression in HK-2 cells treated with 20 μg/mL AAI for 72 h, with or without 1 ng/mL recombinant human SMOC2 protein (rSMOC2). **e** Western blot and quantification of γH2AX expression in HK-2 cells transfected with either Myc-empty vector or Myc-SMOC2 plasmid following treatment with 20 μg/mL AAI. **f** Western blot and quantification of γH2AX expression in HK-2 cells transfected with either scramble siRNA or SMOC2 siRNA following treatment with 20 μg/mL AAI. **g** Western blot and quantification of γH2AX expression in primary mTECs from WT and SMOC2 KO mice treated with 20 μg/mL AAI for 72 h. *n* = 6 per group (in vivo), *n* = 3 per group (in vitro). *p < 0.05, **p < 0.01, ***p < 0.001, ****p < 0.0001

To further assess the protective role of SMOC2 in AAI-induced tubular injury, we examined γH2AX (phosphorylated H2AX at Ser139), a well-established marker of DNA damage [41]. Following AAI administration, the number of γH2AX^+^ cells increased in both WT and SMOC2 KO mice (Fig. 3a). Notably, SMOC2 KO mice exhibited a significantly higher number of γH2AX^+^ cells compared with WT controls (Fig. 3b). This finding was corroborated by Western blot analysis, which revealed markedly elevated γH2AX expression in the kidneys of SMOC2 KO mice after AAI injection (Fig. 3c). To determine whether SMOC2 directly modulates DNA damage in renal tubular cells, HK-2 cells were pretreated with recombinant human SMOC2 (rSMOC2, 1 ng/mL) for 5 h and subsequently exposed to AAI (20 μg/mL) for 72 h. rSMOC2 treatment significantly attenuated AAI-induced γH2AX expression (Fig. 3d). Consistently, SMOC2 overexpression reduced γH2AX levels following AAI treatment (Fig. 3e), whereas siRNA-mediated knockdown of SMOC2 further increased γH2AX expression in HK-2 cells (Fig. 3f). To extend these findings to primary cells, mTECs isolated from SMOC2 WT and KO mice were treated with AAI (20 μg/mL) for 72 h. In agreement with the in vivo data, SMOC2-deficient mTECs displayed significantly increased γH2AX expression compared with WT cells following AAI exposure (Fig. 3g). Collectively, these results demonstrate that SMOC2 deficiency exacerbates AAI-induced DNA damage in renal tubular cells both in vivo and in vitro.

### SMOC2 deficiency exacerbates AAI-induced cellular apoptosis

**Fig. 4:**
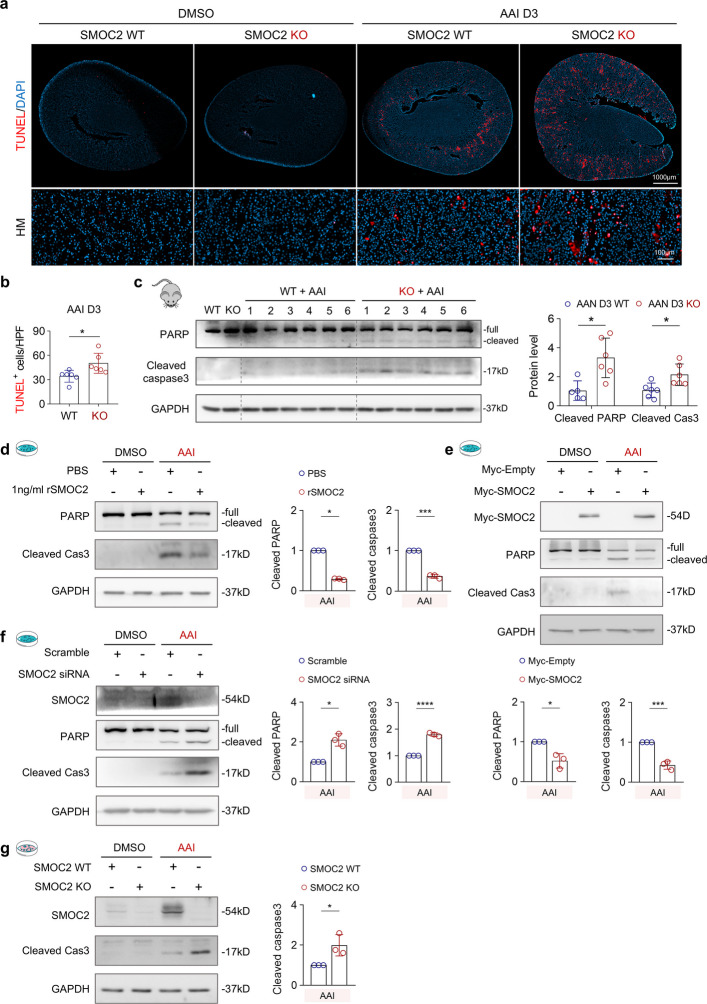
SMOC2 knockout exacerbates cellular apoptosis in male AAN mice and AAI-treated renal tubular cells. **a** Upper panel: Representative images of TUNEL staining, to visualize apoptotic cells, in transverse kidney sections from DMSO- or AAI-treated mice. Lower panel: HM images highlighting TUNEL^+^ cells in each group. **b** Quantification of TUNEL.^+^ cells per HPF in AAI-injected SMOC2 WT and KO mice at day 3. **c** Western blot and quantitative analysis of PARP and cleaved caspase 3 expression in kidney tissues from WT and SMOC2 KO mice on day 3 following DMSO or AAI injection. **d** Western blot and quantification of PARP and cleaved caspase 3 expression in HK-2 cells treated with 20 μg/mL AAI for 72 h, with or without 1 ng/mL rSMOC2. **e** Western blot and quantification of PARP and cleaved caspase 3 expression in HK-2 cells transfected with either Myc-empty or Myc-SMOC2 plasmid following treatment with 20 μg/mL AAI. **f** Western blot and quantification of PARP and cleaved caspase 3 expression in HK-2 cells transfected with either scramble siRNA or SMOC2 siRNA following treatment with 20 μg/mL AAI. **g** Western blot and quantification of cleaved caspase 3 expression in primary mTECs from WT and SMOC2 KO mice treated with 20 μg/mL AAI for 72 h. *n* = 6 per group (in vivo), *n* = 3 per group (in vitro). *p < 0.05, **p < 0.01, ***p < 0.001, ****p < 0.0001

Excessive DNA damage can activate apoptotic pathways [42], and apoptosis has been implicated in tubular loss in AAI-induced AKI [43]. To evaluate tubular apoptosis in vivo, we first performed terminal deoxynucleotidyl transferase dUTP nick-end labeling (TUNEL) staining. Following AAI administration, the number of TUNEL^+^ cells was significantly higher in SMOC2 KO mice than in WT controls (Fig. 4a, b). To further confirm apoptotic activation, key apoptotic markers were analyzed by Western blot. Levels of cleaved PARP and cleaved caspase 3 were markedly increased in the kidneys of SMOC2 KO mice compared with WT mice after AAI injection (Fig. 4c), indicating enhanced apoptosis. In vitro, treatment with rSMOC2 (Fig. 4d) or SMOC2 overexpression (Fig. 4e) significantly attenuated AAI-induced apoptosis, as evidenced by reduced expression of cleaved PARP and cleaved caspase 3. In contrast, SMOC2 knockdown in HK-2 cells (Fig. 4f) or genetic deletion of SMOC2 in mTECs (Fig. 4g) further potentiated AAI-induced apoptotic signaling. Collectively, these results indicate that SMOC2 plays a protective role in limiting AAI-induced tubular epithelial apoptosis in both in vivo and vitro models.

### SMOC2 deficiency worsens cisplatin-induced tubular DNA damage, apoptosis, and tubular injury

To extend our findings to a clinically relevant AKI model, we next examined the role of SMOC2 in cisplatin-induced acute tubular injury, a common cause of drug-induced AKI in clinical settings [37]. Based on previous studies [44], cisplatin doses ranging from 10 to 30 mg/kg BW are commonly used to induce AKI. However, we observed that a single injection of cisplatin at 20 mg/kg BW was lethal in SMOC2 KO mice (Fig. S4a), as demonstrated by survival analysis (Fig. S4b). Indeed, 4 out of 5 SMOC2 KO mice developed severe distress and required euthanasia by day 3 following cisplatin administration. To establish a non-lethal yet injurious dose, we performed dose titration and identified 12.5 mg/kg BW cisplatin as sufficient to induce marked tubular injury without causing mortality by day 3 (Fig. S4c). Consistent with our observations in the AAI-induced AKI model, SMOC2 expression was upregulated in cisplatin-injured kidneys (Fig. S4d). At this dose, cisplatin treatment induced robust DNA damage, as indicated by increased γH2AX expression (Fig. S4d), which was further exacerbated in SMOC2 KO mice (Fig. S4e). Assessment of renal function revealed significantly higher Scr (Fig. S4f) and BUN (Fig. S4g) levels in SMOC2 KO mice compared with WT controls following cisplatin injection. Histological evaluation by HE staining demonstrated markedly aggravated tubular injury in SMOC2 KO kidneys (Fig. S4h, i). Furthermore, γH2AX immunostaining (Fig. S5a, b) and cleaved caspase 3 staining (Fig. S5c, d) confirmed significantly increased DNA damage and apoptosis in SMOC2 KO mice relative to WT mice. Collectively, these findings extend our AAI-induced AKI observations and further support a protective role for SMOC2 in limiting tubular DNA damage, apoptosis, and injury during AKI.

### SMOC2 deficiency promotes fibrosis in AAI-induced CKD by exacerbating tubular injury during AKI

Unlike cisplatin-induced AKI, which is often lethal within days [45] and therefore limits its utility for studying the AKI-to-CKD transition, AAI induces an initial AKI followed by progressive renal fibrosis, providing a robust model to investigate AKI-to-CKD progression [46]. Given that initial tubular injury is a critical determinant of subsequent renal fibrosis [8], we examined whether the protective effect of SMOC2 during AKI extends into the chronic phase (Fig. S6a). At 21 days post-AAI injection, both WT and SMOC2 KO mice exhibited significantly elevated Scr (Fig. S6b) and BUN (Fig. S6c) levels, with an even greater increase in SMOC2 KO AAN mice. Histological analysis revealed a pronounced loss of intact tubules in SMOC2 KO AAN mice at day 21 (Fig. S6d, e), which was further supported by reduced Villin 1 staining (Fig. S6f), indicating a loss of mature tubular epithelial cells. In line with the role of tubular injury as a driver of fibrosis, Masson's trichrome staining demonstrated significantly increased fibrosis in SMOC2 KO mice compared to WT (Fig. S6d, lower panel). Moreover, immunofluorescence staining revealed increased expression of fibrotic markers, including fibronectin and α-smooth muscle actin (α-SMA), at 21 days after AAI injection, with a more pronounced increase in SMOC2 KO mice (Fig. S7a). These observations were further corroborated by Western blot analysis, which showed higher levels of fibronectin, collagen I, and α-SMA in SMOC2 KO kidneys relative to WT kidneys following AAI injection (Fig. S7b). Collectively, these findings indicate that the protective effect of SMOC2 during the acute phase of kidney injury persists into the chronic stage, thereby attenuating maladaptive repair and limiting fibrosis during AKI-to-CKD progression.

### Transcriptomic analysis reveals aberrant cell cycle activation in SMOC2-deficient AAN kidneys

**Fig. 5:**
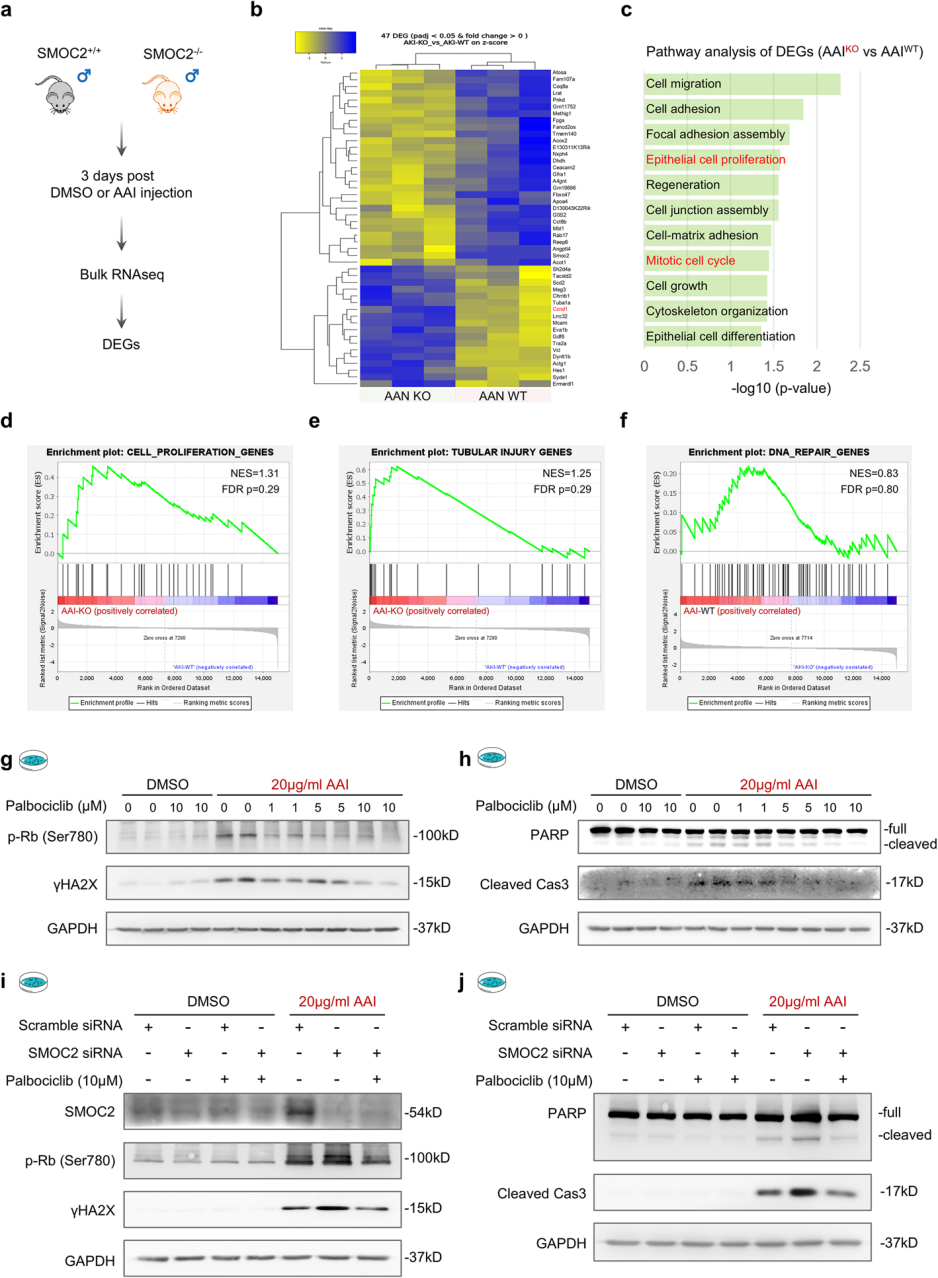
Transcriptomic analysis reveals an inverse association between SMOC2 expression and cellular proliferation gene signature. **a** Total RNA was extracted from kidneys of male WT and SMOC2 KO mice injected with DMSO or AAI and subjected to bulk RNA sequencing. (*n* = 3 per group) **b** Heatmap visualization of differentially expressed genes (DEGs) between AAN WT and KO mice. **c** Pathway analysis of DEGs highlights a strong association with cell cycle regulation and cellular proliferation. **d**-**f** Gene Set Enrichment Analysis (GSEA) comparing bulk RNA-seq profiles of AAN WT and KO mice using literature-curated gene sets (Supplementary Table 2) associated with cellular proliferation, tubular injury, and DNA repair. **g** Western blot analysis of phosphorylated Rb (p-Rb Ser780) and γH2AX expression in HK-2 cells treated with DMSO or 20 μg/mL AAI for 72 h in the presence of increasing doses of the CDK4/6 inhibitor palbociclib. **h** Western blot analysis of PARP and cleaved caspase 3 expression in HK-2 cells treated with DMSO or 20 μg/mL AAI for 72 h in the presence of increasing doses of the CDK4/6 inhibitor palbociclib. **i** Western blot analysis of SMOC2, p-Rb and γH2AX expression in HK-2 cells treated with DMSO or AAI for 72 h, with or without palbociclib, in the presence or absence of SMOC2 siRNA. **j** Western blot analysis of PARP and cleaved caspase 3 expression in HK-2 cells treated with DMSO or AAI for 72 h, with or without palbociclib, in the presence or absence of SMOC2 siRNA. *n* = 3 per group

To explore the molecular mechanisms underlying the aggravated injury observed in SMOC2 KO mice, we performed transcriptomic analysis of kidneys from WT and KO mice on day 3 following DMSO or AAI injection (Fig. 5a). This analysis identified a set of differentially expressed genes (DEGs) between WT and SMOC2 KO kidneys under AAN conditions (Fig. 5b). Gene Ontology (GO) enrichment analysis revealed that the majority of DEGs were significantly enriched in biological processes related to cell proliferation and cell cycle regulation (Fig. 5c). Consistently, gene set enrichment analysis (GSEA) demonstrated a strong positive enrichment of cell proliferation-associated gene sets in SMOC2 KO AAN kidneys (Fig. 5d). In parallel, GSEA revealed that SMOC2 KO mice exhibited increased enrichment of tubular injury-associated gene signatures (Fig. 5e), accompanied by a negative enrichment of DNA repair-related gene sets (Fig. 5f). Together, these transcriptomic findings are consistent with our in vivo observations and indicate that SMOC2 deficiency is associated with aberrant activation of cell proliferation programs, exacerbated tubular injury, and impaired DNA repair during AAI-induced AKI.

### Pharmacological induction of G1 arrest mitigates DNA damage and apoptosis in AAI-induced AKI

After kidney injury, dedifferentiation and proliferation of residual TECs are critical for tubular regeneration [10]. However, accumulating evidence indicates that during the acute phase of AKI, transient cell cycle arrest provides additional time for DNA repair and thereby limits renal injury across multiple AKI models [47–53]. This framework may explain why enrichment of cell proliferation pathways in SMOC2-deficient AAN mice is paradoxically associated with worsened kidney injury, as excessive or premature cell cycle re-entry during the acute phase may bypass protective cell cycle arrest required for adequate DNA repair, ultimately exacerbating renal damage. As the role of G1-phase arrest in AAI-induced AKI has not been specifically examined, we first investigated this in vitro using palbociclib, an FDA-approved CDK4/6 inhibitor that induces G1 arrest [54]. In HK-2 cells, palbociclib (10 μM) markedly reduced AAI-induced phosphorylation of retinoblastoma protein at Ser780 (p-Rb Ser780), a readout of CDK4/6 activity (Fig. 5g). This reduction was accompanied by decreased DNA damage and apoptosis following AAI exposure (Fig. 5g, h). Moreover, SMOC2 knockdown further increased AAI-induced DNA damage and apoptosis in HK-2 cells (Fig. 5i, j), both of which were effectively rescued by palbociclib treatment. These results indicate that pharmacological induction of G1 arrest protects TECs from AAI-induced DNA damage and apoptosis in vitro.

**Fig. 6:**
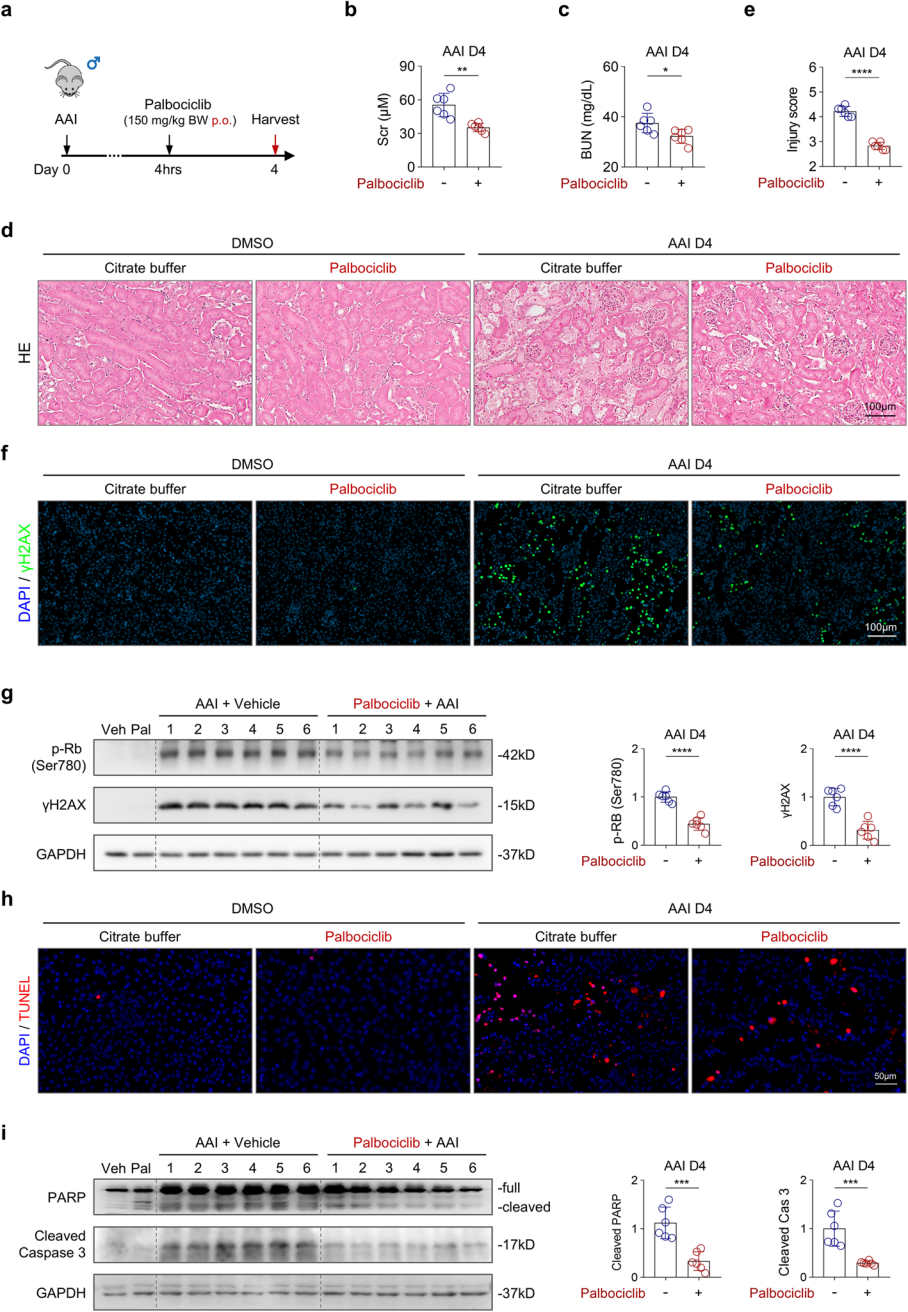
Post-injury treatment with palbociclib reduces DNA damage, apoptosis, and tubular injury in male AAN mice. **a** Schematic diagram illustrating the post-injury treatment protocol with palbociclib in the AAN male mice. **b, c** Scr and BUN levels in AAN mice treated with palbociclib or vehicle (citrate buffer). **d** Representative HE-stained kidney sections from DMSO- or AAI-treated mice following treatment with either palbociclib or citrate buffer. **e** Quantification of tubular injury scores in AAN mice treated with palbociclib or citrate buffer. **f** Representative γH2AX staining in kidney sections from DMSO- or AAI-treated mice following treatment with either palbociclib or citrate buffer. **g** Western blot analysis and quantification of p-Rb (Ser780) and γH2AX expression in kidney tissues from DMSO- or AAI-treated mice administered with palbociclib or citrate buffer. **h** Representative TUNEL staining in kidney sections from DMSO- or AAI-treated mice following treatment with either palbociclib or citrate buffer. **i** Western blot and quantification analysis of PARP and cleaved caspase 3 expression in kidney tissues from DMSO- or AAI-treated mice following treatment with either palbociclib or citrate buffer. *n* = 4 for the DMSO + citrate group, *n* = 5 for the DMSO + palbociclib group, and *n* = 6 for the AAN + citrate and AAN + palbociclib groups. *p < 0.05, **p < 0.01, ***p < 0.001, ****p < 0.0001

To validate these findings in vivo, AAN mice were pretreated with palbociclib (Fig. S8a). Palbociclib pre-treatment effectively inhibited CDK4/6 activity, as evidenced by reduced p-Rb expression (Fig. S8g), and resulted in a modest reduction in Scr levels (Fig. S8b), although BUN levels were unchanged (Fig. S8c). Nevertheless, palbociclib pre-treatment significantly alleviated tubular injury (Fig. S8d, e), DNA damage (Fig. S8f, g), and apoptosis (Fig. S8h, i). Given that prophylactic treatment is not clinically practical, we next evaluated the therapeutic potential of G1 arrest following injury onset. To this end, palbociclib was administered 4 h after AAI injection (Fig. 6a). Notably, therapeutic administration conferred even greater renal protection, as demonstrated by significant reductions in both Scr and BUN levels (Fig. 6b, c) and a lower tubular injury score compared with pretreatment (post-treatment: 2.83 ± 0.15 vs pretreatment: 3.33 ± 0.28; Fig. 6d, e). Consistent with pre-treatment results, palbociclib significantly reduced p-Rb expression (Fig. 6g), DNA damage (Fig. 6f, g), and apoptosis (Fig. 6h, i). These protective effects were accompanied by a reduced number of Ki-67⁺ proliferating tubular cells (Fig. S9), suggesting that suppression of aberrant cell cycle progression limits further tubular injury. To define the latest effective therapeutic window, palbociclib was administered up to 24 h after AAI injection (Fig. S10a). At this time point, palbociclib continued to inhibit Rb phosphorylation (Fig. S10f) and showed a trend toward renal protection, as reflected by reduced Scr (Fig. S10b), BUN (Fig. S10c), and tubular injury scores (Fig. S10d, e), although these changes did not reach statistical significance. In contrast, γH2AX expression remained significantly reduced in palbociclib-treated AAN mice (Fig. S10f). These findings indicate that while palbociclib retains partial efficacy when administered 24 h after injury, its protective effects are substantially diminished compared with treatment initiated before or shortly after injury onset. Collectively, these results demonstrate that pharmacological induction of G1 cell cycle arrest mitigates DNA damage and apoptosis in AAI-induced AKI and highlight a limited but therapeutically relevant time window during which CDK4/6 inhibition confers renal protection.

### Loss of SMOC2 drives aberrant cell cycle progression through activation of CCND1-CDK4/6 signaling

**Fig. 7:**
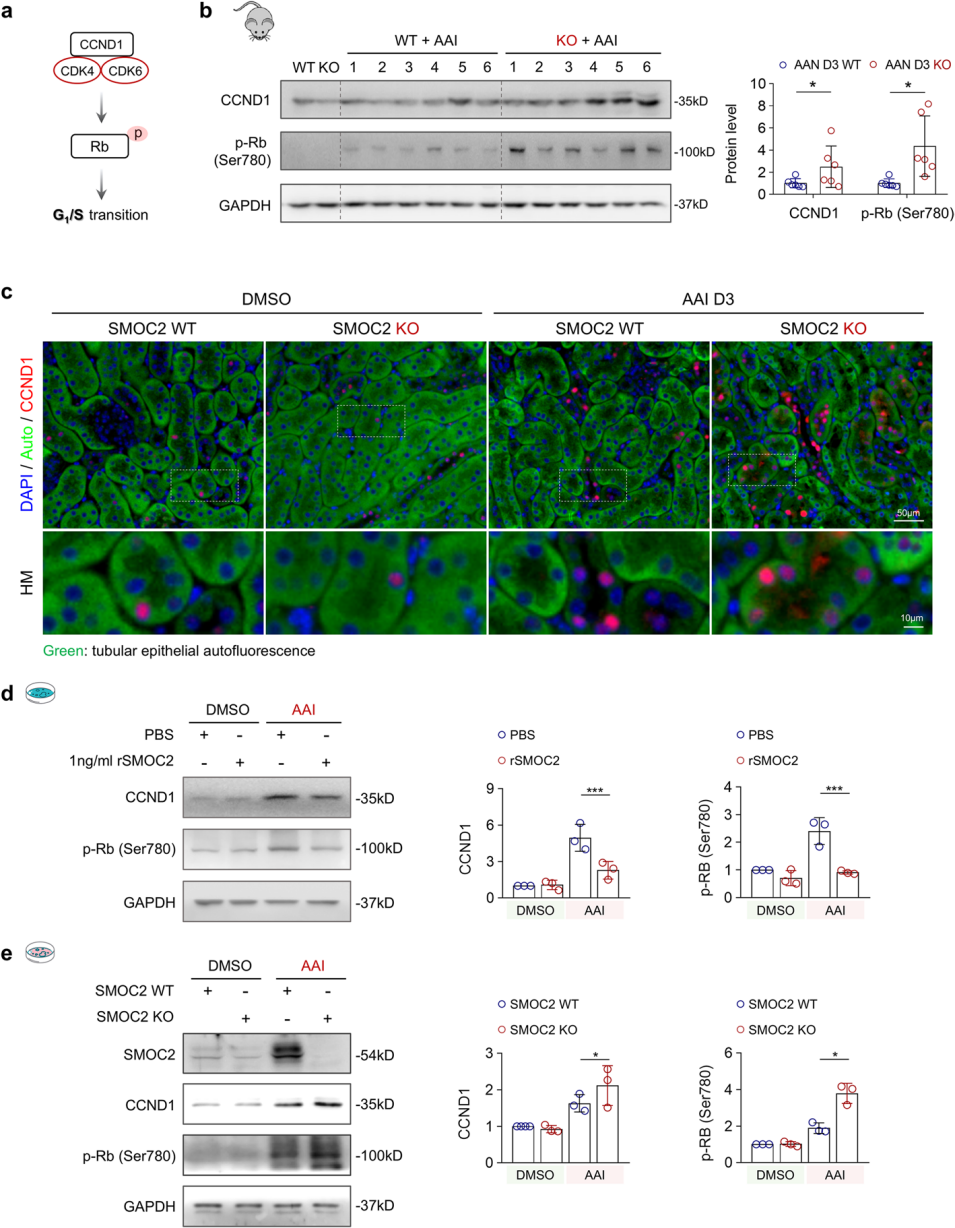
SMOC2 knockout leads to CCND1 upregulation, which aggravates Rb phosphorylation at Ser780 via the CDK4/6 pathway. **a** Schematic diagram illustrating the interaction of CCND1 with the CDK4/6 complex, leading to Rb phosphorylation at Ser780. This pathway plays a critical role in G1/S progression. **b** Western blot analysis and quantification of CCND1 and p-Rb Ser780 expression in kidney tissues from SMOC2 WT or KO mice subjected to DMSO or AAI. **c** Representative immunofluorescence images of CCND1 with epithelial autofluorescence in kidney sections from SMOC2 WT or KO mice treated with DMSO or AAI. **d** Western blot analysis and quantification of CCND1 and p-Rb Ser780 expression in HK-2 cells treated with 20 μg/mL AAI for 72 h, with or without 1 ng/mL rSMOC2. **e** Western blot analysis and quantification of CCND1 and p-Rb Ser780 expression in primary mTECs isolated from WT and SMOC2 KO mice treated with 20 μg/mL AAI for 72 h. *n* = 6 per group (in vivo), *n* = 3 per group (in vitro). *p < 0.05, **p < 0.01, ***p < 0.001, ****p < 0.0001

To elucidate the mechanism by which SMOC2 restrains cell cycle progression, we examined the involvement of the CDK4/6 pathway. Among the DEGs identified between WT and SMOC2 KO AAN kidneys, CCND1 mRNA was significantly upregulated in SMOC2 KO mice (Fig. 5b). CCND1 encodes cyclin D1, which associates with CDK4/6 to phosphorylate Rb at Ser780, thereby releasing E2F transcription factors that drive the expression of genes required for G1/S phase transition (Fig. 7a) [55, 56]. Consistent with this mechanism, CCND1 expression was increased in WT AAN kidneys and further elevated in SMOC2 KO kidneys, accompanied by enhanced Rb phosphorylation, indicating increased CDK4/6 activity (Fig. 7b). Immunofluorescence staining further demonstrated that CCND1 expression was predominantly localized to renal tubules, with significantly more CCND1^+^ TECs observed in SMOC2 KO AAN kidneys (Fig. 7c). To determine whether SMOC2 directly modulates this pathway, HK-2 cells were treated with rSMOC2, which significantly attenuated AAI-induced CCND1 expression and Rb phosphorylation (Fig. 7d). Conversely, genetic deletion of SMOC2 in primary mTECs further exacerbated AAI-induced upregulation of CCND1 and p-Rb (Fig. 7e). Cell cycle analysis demonstrated that rSMOC2 treatment promoted accumulation of cells in the G1 phase, accompanied by a reduction in S-phase entry in AAI-treated HK-2 cells (Fig. S11), indicating delayed G1/S transition under DNA damage conditions. Collectively, these results demonstrate that SMOC2 restrains aberrant cell cycle progression by suppressing CCND1-CDK4/6 pathway activation, thereby preventing inappropriate G1/S transition and mitigating AAI-induced tubular injury.

### SMOC2 interacts with integrin β3 to inhibit ERK-mediated upregulation of CCND1

**Fig. 8:**
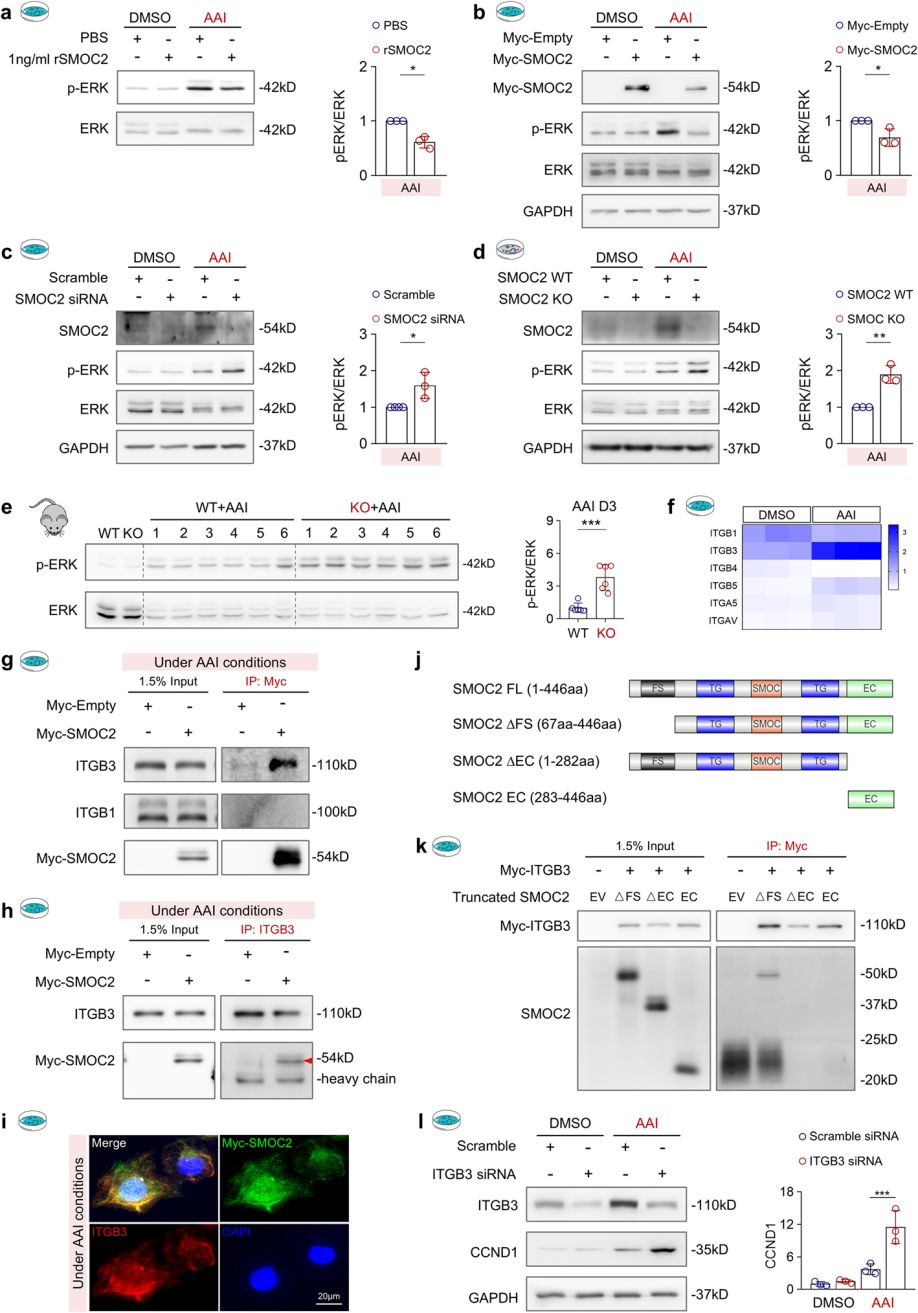
SMOC2 knockout enhances ERK activation, leading to CCND1 upregulation. **a** Western blot analysis and quantification of phosphorylated ERK (p-ERK) and total ERK expression in HK-2 cells treated with 20 μg/mL AAI for 72 h, with or without 1 ng/mL rSMOC2. **b** Western blot analysis and quantification of p-ERK and total ERK expression in HK-2 cells transfected with either Myc-empty or Myc-SMOC2 plasmid following treatment with 20 μg/mL AAI. **c** Western blot analysis and quantification of p-ERK and total ERK expression in HK-2 cells transfected with either scramble siRNA or SMOC2 siRNA following treatment with 20 μg/mL AAI. **d** Western blot and quantification analysis of p-ERK and total ERK expression in primary mTECs isolated from WT and SMOC2 KO mice treated with 20 μg/mL AAI for 72 h. **e** Western blot and quantification analysis of p-ERK and total ERK expression in kidney tissues from WT and SMOC2 KO mice subjected to DMSO or AAI. **f** Heatmap visualization of differentially expressed integrins in HK-2 cells treated with DMSO or AAI for 48 h. **g** Co-immunoprecipitation (Co-IP) analysis under AAI treatment using Myc-beads to pull down Myc-SMOC2 from HK-2 cell lysates transfected with either Myc-empty or Myc-SMOC2. Western blot analysis was performed to detect the interaction between SMOC2 and integrins β1 (ITGB1) or β3 (ITGB3). **h** Reciprocal Co-IP analysis under AAI treatment using an ITGB3 antibody to pull down ITGB3 from HK-2 cell lysates transfected with either Myc-empty or Myc-SMOC2. Western blot analysis was performed to detect the interaction between ITGB3 and Myc-SMOC2. **i** Immunofluorescence staining of HK-2 cells under AAI treatment, showing Myc-SMOC2 (green) and ITGB3 (red), with yellow regions indicating colocalization of Myc-SMOC2 and ITGB3. **j** Schematic representation of full-length (FL) and truncated SMOC2 constructs used for domain mapping. SMOC2 FL (1–446 aa), SMOC2 ΔFS (67–446 aa, lacking the FS domain), SMOC2 ΔEC (1–282 aa, lacking the EC domain), and SMOC2 EC (283–446 aa, EC domain only). **k** Co-IP analysis using Myc-beads to pull down Myc-ITGB3 from HK-2 cell lysates co-transfected with Myc-ITGB3 and truncated SMOC2 plasmids. Western blot analysis was performed to detect the interaction between SMOC2 and ITGB3. A homemade SMOC2 antibody (a generous gift from Dr. Ursula) was used to detect all truncated SMOC2 proteins. **l** Western blot analysis and quantification of ITGB3 and CCND1 expression in HK-2 cells transfected with either scramble siRNA or ITGB3 siRNA following treatment with 20 μg/mL AAI. *n* = 6 per group (in vivo), *n* = 3 per group (in vitro). *p < 0.05, **p < 0.01, ***p < 0.001, ****p < 0.0001

To elucidate how SMOC2 regulates CCND1 expression, we focused on the ERK signaling pathway, a well-established upstream regulator of CCND1 transcription [57] and a key contributor to kidney injury in multiple AKI models [58, 59]. In HK-2 cells, treatment with rSMOC2 (Fig. 8a) or SMOC2 overexpression (Fig. 8b) markedly attenuated AAI-induced ERK phosphorylation (p-ERK). In contrast, SMOC2 knockdown in HK-2 cells (Fig. 8c) or genetic deletion of SMOC2 in mTECs (Fig. 8d) further potentiated ERK activation in response to AAI. Consistently, in vivo analysis revealed increased p-ERK levels in AAN WT kidneys, with a further elevation observed in SMOC2 KO kidneys (Fig. 8e), indicating that SMOC2 negatively regulates ERK pathway activation during AAI-induced injury.

Given that SMOC2 primarily signals through integrin receptors [17, 25, 26, 30], we next sought to identify the integrin mediating this effect. Expression profiling of integrins in DMSO- and AAI-treated HK-2 cells revealed that integrin β1 (ITGB1) and integrin β3 (ITGB3) were the most abundantly expressed β integrins (Fig. 8 and Fig. S12a), both of which have been reported as SMOC2-interacting partners and are expressed in injured kidneys [60]. Interestingly, AAI treatment significantly decreased ITGB1 expression while increasing ITGB3 expression (Fig. 8f), suggesting a potential functional role for ITGB3 in SMOC2-mediated signaling. To determine whether SMOC2 physically interacts with ITGB1 or ITGB3, we performed co-immunoprecipitation (Co-IP) assays in AAI-treated HK-2 cells (Fig. 8g). These assays revealed a specific interaction between SMOC2 and ITGB3, but not ITGB1. This interaction was further confirmed by reciprocal Co-IP using endogenous ITGB3 (Fig. 8h) and by co-immunostaining of Myc-tagged SMOC2 with ITGB3 (Fig. 8i), which demonstrated their colocalization along the cell membrane. Structurally, SMOC2 comprises a SMOC-specific acidic domain flanked by two TG domains that separate the FS and EC domains [19] (Fig. 8j). To define the region responsible for the SMOC2-ITGB3 interaction, we performed Co-IP assays using a series of truncated SMOC2 constructs co-transfected with ITGB3 (Fig. 8k). Deletion of the FS domain (ΔFS) did not affect ITGB3 binding, whereas deletion of the EC domain (ΔEC) completely abolished the interaction. Surprisingly, expression of the EC domain alone was insufficient to mediate binding (Fig. 8k). Together, these findings indicate that SMOC2-ITGB3 interaction requires the cooperative contribution of multiple structural domains, rather than a single binding motif.

Finally, to assess the functional significance of the SMOC2-ITGB3 interaction, we knocked down ITGB3 in AAI-treated HK-2 cells. ITGB3 depletion led to significantly enhanced ERK phosphorylation (Fig. S12b), accompanied by increased CCND1 expression (Fig. 8l). Collectively, these findings indicate that SMOC2 interacts with ITGB3 to suppress ERK-mediated CCND1 upregulation, thereby restraining cell cycle progression and protecting against AAI-induced tubular injury.

### Recombinant SMOC2 protein treatment protects against AAI-induced AKI

SMOC2, a 54-kDa matricellular protein, is detectable in the serum and urine of patients with kidney disease [61], suggesting that glomerular permeability allows its passage and supporting its potential as a recombinant protein therapeutic. To explore this possibility, we examined whether systemic administration of rSMOC2 confers protection against AAI-induced AKI. In a pilot study, using γH2AX as a readout of DNA damage, we found that daily administration of rSMOC2 at a dose of 200 ng/kg BW significantly reduced γH2AX expression in AAI-injected mice at day 4 (Fig. S13a, b).

Upon expanding the cohort size (Fig. S14a), rSMOC2 treatment modestly improved Scr levels (Fig. S14b) and significantly reduced BUN levels compared with PBS-treated controls (Fig. S14c). Consistent with improved renal function, rSMOC2 markedly alleviated tubular injury (Fig. S14d), as reflected by lower tubular injury scores (Fig. S14e), reduced DNA damage (Fig. S14f, h, j), and decreased apoptosis (Fig. S14g, k).

In line with our in vitro findings, rSMOC2 treatment suppressed ERK activation and CCND1 expression, accompanied by reduced phosphorylation of Rb, as demonstrated by Western blot analysis (Fig. S14h–j), indicating attenuation of aberrant cell cycle progression in vivo.

Given that both AAI [62] and SMOC2 [28] have been reported to affect the liver, we next evaluated potential off-target effects of rSMOC2 treatment in this organ. Histological analysis confirmed that the AAI dose used did not induce overt hepatic injury, as assessed by HE staining (Fig. S15a). At the molecular level, only minimal hepatic DNA damage was detected, characterized by sparse γH2AX^+^ cells (Fig. S15b), and importantly, this subtle DNA damage did not lead to apoptosis, as indicated by the absence of cleaved caspase 3 staining (Fig. S15c). Under these conditions, rSMOC2 treatment did not induce any detectable difference in the liver (Fig. S15), indicating that its protective effects are largely restricted to the kidney when other organs are minimally affected. Collectively, these results demonstrate that recombinant SMOC2 administration mitigates AAI-induced tubular injury by reducing DNA damage, apoptosis, and aberrant cell cycle progression, thereby supporting the therapeutic potential of rSMOC2 for the treatment of AKI.

## Discussion

**Fig. 9:**
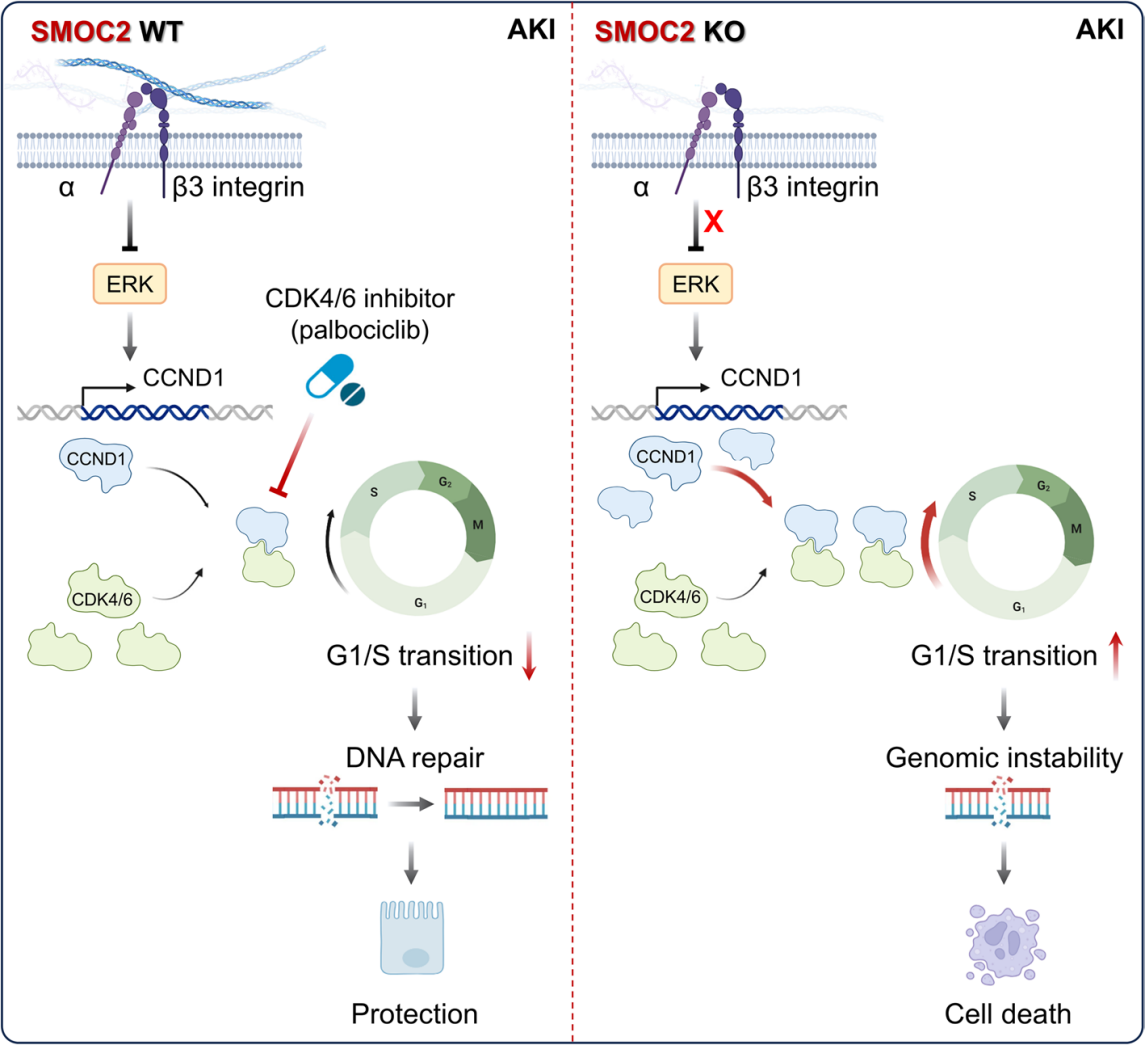
Schematic model illustrating the protective role of SMOC2 in tubular injury following AKI. After AKI, tubular epithelial cells upregulate and secrete the matricellular protein SMOC2, which binds to ITGB3 on the tubular cell surface. This interaction suppresses ERK signaling, leading to transcriptional downregulation of CCND1 and reduced CDK4/6 activity. Consequently, phosphorylation of Rb at Ser780 is decreased, resulting in G0/G1 cell cycle arrest. By restraining G1/S transition, SMOC2 facilitates DNA repair and prevents apoptosis in injured tubular epithelial cells. Pharmacological inhibition of CDK4/6 with palbociclib phenocopies the protective effect of SMOC2 by enforcing G1 arrest, thereby limiting DNA damage and cell death. In contrast, loss of SMOC2 leads to unchecked ERK activation, increased CCND1-CDK4/6 signaling, accelerated G1/S transition, genomic instability, and tubular cell death. Together, these findings identify SMOC2 as a critical regulator of tubular injury and repair following AKI

In this study, we investigated the role of SMOC2 in tubular injury following AKI and its contribution to CKD transition. By employing two tubular-specific injury models, we were able to distinguish the effects of SMOC2 on renal TECs from its previously described actions on fibroblasts. We demonstrate that SMOC2 deficiency exacerbates acute tubular injury and accelerates the progression from AKI to CKD. Mechanistically, SMOC2 interacts with ITGB3 to inhibit ERK activation, thereby suppressing CCND1/CDK4/6-mediated G1/S cell cycle progression. This SMOC2-mediated G1 arrest provides injured TECs with additional time for DNA repair, resulting in reduced DNA damage and apoptosis (Fig. 9). Together, these findings uncover a previously unrecognized protective role of SMOC2 during AKI that is distinct from its pro-fibrotic effects in CKD.

This biphasic function, protective during the acute phase yet potentially profibrotic later, is reminiscent of other MCPs, such as tenascin-C [63–66] and periostin [67–70], which preserve tubular integrity during AKI but drive fibrosis in CKD. Our results reinforce the concept that MCP function in kidney disease is highly time-dependent. We acknowledge that inducible deletion of SMOC2 during the chronic phase (e.g., using tamoxifen-inducible SMOC2 flox/flox mice) will provide more direct evidence for its late-stage roles, and we are currently generating these models to address this question in future studies.

Transcriptomic profiling of WT and SMOC2 KO AAN kidneys revealed significant enrichment of cell cycle-related pathways, with proliferation-associated genes markedly upregulated in SMOC2-deficient mice. At first glance, this finding appears paradoxical, as cellular proliferation is generally regarded as essential for tubular regeneration following AKI [10], and is therefore often used as a surrogate marker of renoprotection [71]. However, widespread and immediate parenchymal cell dedifferentiation and/or proliferation may be counterproductive, as they imply a transient loss of specialized cellular functions. If the remaining TECs, which sustain the minimal residual kidney function, were to undergo extensive mitosis, it could further impair functional performance, potentially resulting in critical failure or even death [72]. This framework provides a plausible explanation for the worsened renal outcomes observed in AAI- and cisplatin-injured SMOC2-deficient mice. In this context, the proliferation signature likely reflects injury severity rather than effective tubular regeneration.

Consistent with this interpretation, we demonstrate that SMOC2-mediated G1 phase cell cycle arrest contributes to its protective effect in AKI, as cells in the G0/G1 phase are more resistant to genotoxic stress than those entering S phase [73]. Pharmacological induction of G1 arrest with palbociclib phenocopied the protective effects observed with SMOC2 in AKI. These findings align with previous studies demonstrating that early and transient cell cycle arrest facilitates AKI recovery by preventing premature cell cycle progression and allowing sufficient time for DNA repair [47–53]. Importantly, however, prolonged cell cycle arrest can lead to cellular senescence [11], leading to inflammation and fibrosis through the senescence-associated secretory phenotype (SASP) [74]. Thus, a delicate balance between transient arrest and timely cell cycle re-entry is critical for successful tubular repair and prevention of fibrosis.

To elucidate the molecular basis of SMOC2-mediated G1 arrest, our transcriptomic and in vitro analyses identify CCND1 as a key downstream effector. CCND1 upregulation has been implicated in AKI pathogenesis: tubule-specific CCND1 deletion mitigates AKI (2023-ASN poster, TH-PO107), while CCND1 overexpression increases susceptibility to ischemic injury [75]. Our findings suggest that SMOC2 delays G1/S phase transition by downregulating CCND1 expression, thereby prolonging the G1 phase to facilitate DNA repair. Additionally, it has been demonstrated that CCND1 overexpression disrupts nucleotide excision repair (NER) specifically in S-phase cells [76], a crucial DNA repair mechanism responsible for removing AAI- and cisplatin-induced DNA adducts [77]. This evidence further supports our finding that SMOC2-mediated CCND1 suppression is protective in AKI.

SMOC2 primarily signals through integrin receptors [17, 25, 26, 30]. Previous work has shown that SMOC2 interacts with ITGB1 in fibroblasts to promote myofibroblast activation and renal fibrosis during CKD [30]. In contrast, our study demonstrates that during AKI, SMOC2 preferentially interacts with ITGB3 in TECs, leading to G1-phase cell cycle arrest and enhanced cell survival. This receptor-dependent signaling underscores a cell type-specific mechanism governing SMOC2 function. Notably, whereas ITGB1-mediated signaling activates ERK in fibro-adipogenic progenitors in aging muscle [78], SMOC2-ITGB3 engagement suppresses ERK activation in TECs during AKI. Although the precise molecular basis of this differential regulation remains unclear, this receptor-dependent behavior mirrors that of periostin, which activates FAK signaling through ITGB3 in podocytes [68] but inhibits FAK signaling via ITGB1 in TECs [69]. Together, these findings suggest that the biological effects of matricellular proteins are dictated by both receptor usage and cellular context.

In summary, our study identifies SMOC2 as a critical regulator of tubular injury in AKI, acting through ITGB3-dependent inhibition of ERK signaling and CCND1-mediated G1/S transition. These findings provide new insight into the stage-specific functions of SMOC2 and highlight early cell cycle regulation as a promising therapeutic target to prevent AKI-to-CKD progression. Future studies employing inducible SMOC2 knockout models will be essential for defining its temporal roles in tubular repair and fibrosis. Nevertheless, the use of global SMOC2 knockout mice remains advantageous, as SMOC2 is a secreted matricellular protein; complete systemic deletion avoids confounding effects from extra-renal sources, particularly given its established roles in lung [27] and liver fibrosis [28].

## Materials and methods

### Generation of SMOC2 knockout mouse

Smoc2tm1.1 (KOMP) Vlcg was generated by the Knockout Mouse Phenotyping Program (KOMP2) at The Jackson Laboratory as we previously described [17]. Briefly, the ZEN-UB1 Velocigene cassette was inserted into the target gene, replacing all coding exons and intervening sequences. Cryo-archived sperm (MMRRC Stock #: 049781-UCD) was obtained from the Mutant Mouse Resource and Research Center (MMRRC) at The Jackson Laboratory and used for in vitro fertilization (IVF) at the Institut de recherche en immunologie et en cancérologie (IRIC). Genotyping was performed using specific primers (Supplemental Table 1). SMOC2 KO mice were compared with WT littermates from the same colony. All C57BL/6 J mice used in this study were originally purchased from The Jackson Laboratory and subsequently bred and maintained in the animal facility of the Centre de Recherche de l’Hôpital Maisonneuve-Rosemont (CRHMR) under specific pathogen-free (SPF) conditions, with ad libitum access to food and water.

### Aristolochic acid nephropathy model

Aristolochic acid nephropathy (AAN) was induced in male C57BL/6 J mice (8–12 weeks old) via a single intraperitoneal (i.p.) injection of aristolochic acid I (AAI, 5 mg/kg BW, Sigma, A5512), as previously described [38]. In female C57BL/6 J mice (8–12 weeks old), AAN was induced using either a single i.p. injection of AAI at 5 or 10 mg/kg BW or daily injections at a dose of 10 mg/kg BW. Mice were sacrificed on either day 3 or day 7 to study AKI, and on day 21 to investigate the transition from AKI to CKD. AAI was dissolved in DMSO at a stock concentration of 10 mg/mL, stored at −20 °C, and diluted with PBS before use. Control mice received an equivalent volume of DMSO diluted in PBS. Mice were euthanized under isoflurane anesthesia, and blood and kidney samples were collected. A portion of the kidney was fixed in 10% formalin for 24–48 h, then embedded in paraffin for histological and immunofluorescence staining. Additional kidney tissue was flash-frozen in liquid nitrogen for quantitative polymerase chain reaction (qPCR) and Western blot analysis. Blood was collected in Serum Gel microtubes (Cat No. 41.1378.005, Sarstedt, Germany), allowed to clot at room temperature for ~ 30 min, and centrifuged at 10,000 × g for 5 min. The serum supernatant was aliquoted and stored at −80 °C.

### Cisplatin-induced AKI model

Mice were administered a single i.p. injection of cisplatin (HY-17394, MedChemExpress) at doses of 10, 12.5, or 20 mg/kg BW. Cisplatin was dissolved in DMF at a stock concentration of 10 mg/mL, stored at −20 °C, and diluted with 0.9% saline before administration. Control mice received an equivalent volume of DMF and 0.9% saline. All mice were sacrificed on day 3, and sample collection was performed as described for the AAN model.

### Isolation and culture of primary mTECs

Primary mTECs were isolated from the renal cortices of SMOC2 WT and KO mice (4–8 weeks of age) under sterile conditions using a collagenase-based digestion protocol adapted from previously published methods [79]. Briefly, kidneys were harvested, and cortical regions were dissected and finely chopped into approximately 1 mm^3^ pieces in ice-cold PBS. Tissue fragments were enzymatically dissociated in collagenase II (2 mg/mL in PBS) at 37 °C for approximately 30 min, with gentle trituration every 10 min to enhance tissue dispersion. Enzymatic activity was terminated by the addition of an equal volume of stop solution containing 5% FBS in PBS. The resulting suspension was passed sequentially through 250 μm and 70 μm mesh filters. Proximal tubule (PT) fragments retained on the 70 μm sieve were recovered by reverse flushing with prewarmed PBS and pelleted by centrifugation at 300 × g for 5 min. After washing, the pellet was resuspended in primary mTEC culture medium composed of DMEM/F12 supplemented with 5% heat-inactivated FBS, 15 mM HEPES, 4 μg/mL dexamethasone, 0.7% ITS Premix (Corning, Cat. No. 354351), 0.25 mM sodium pyruvate, 0.05 mM L-ascorbic-2-phosphate, penicillin (100 IU/mL), and streptomycin (100 μg/mL). Isolated tubule fragments were plated and maintained under static conditions for 48 h at 37 °C in a humidified incubator (95% air, 5% CO₂). The culture medium was replaced for the first time at 48 h and subsequently refreshed every two days. By approximately day 7, cells formed a confluent epithelial monolayer, defined as passage 0. For all in vitro experiments, only passage 1 mTECs were used. mTECs were characterized by the expression of epithelial markers E-cadherin [80] and cytokeratin-18 (CK18) [81], and negative for mesenchymal markers α-SMA and vimentin, immune cell marker CD45, and endothelial cell marker CD31. The purity of TECs was confirmed by immunofluorescence, showing that more than 95% of cells were positive for the epithelial markers E-cadherin or CK18 (Fig. S16). In addition, renal tubules isolated from naïve and AAN-treated mice were directly processed for Western blot or qPCR analysis.

### Data statistics and analysis

Data are expressed as mean ± standard deviation. Statistical significance was assessed by a two-tailed Student’s t-test for two-group comparisons or a one-way ANOVA for multigroup comparisons followed by a Tukey post hoc test for subgroup comparisons. *P* < 0.05 was considered significant. Statistical analysis and graphical representation were performed using GraphPad Prism version 8.0.1. Additional details for methods and materials are provided in the Supplementary Methods and Materials.

## Supplementary Information

Supplementary material 1.

Supplementary material 2.

## Data Availability

All data generated or analyzed in this study were included in the main text and the Supplementary Material for this article.
