# Supplementary material for: Matricellular protein SMOC2 safeguards tubular integrity in acute kidney injury via integrin β3-dependent inhibition of CCND1-CDK4/6 axis
Source: Mol Biomed. 2026 Feb 10;7:11. doi: 10.1186/s43556-026-00407-6 (PMC12886620; doi:10.1186/s43556-026-00407-6)
Supplement: Supplementary file 1 — Supplementary material 1. [file 43556_2026_407_MOESM1_ESM.docx]

Matricellular protein SMOC2 safeguards tubular integrity in acute kidney injury via integrin β3-dependent inhibition of CCND1-CDK4/6 axis

Peng Gao^1,2^, Schrodinger Cenatus^2,3^, Dan Zhang^4^, Siwei Chu^2^, Nathalie Henley^2^, Vincent Pichette^1,2,5^, Jonatan Barrera-Chimal^2^, Casimiro Gerarduzzi^1,2,3,5^*

^1^Department of Pharmacology and Physiology, Faculty of Medicine, University of Montreal, Montreal, Quebec, Canada

^2^Maisonneuve-Rosemont Hospital Research Center, Center affiliated with the University of Montreal, Montreal, Quebec, Canada

^3^Department of Biochemistry and Molecular Medicine, Faculty of Medicine, University of Montreal, Montreal, Quebec, Canada

^4^Department of Nephrology, Affiliated hospital of Xuzhou Medical University, Xuzhou, China

^5^Department of Nephrology, Maisonneuve-Rosemont Hospital, Montreal, Quebec, Canada

**Emails:**

Peng Gao: peng.gao@umontreal.ca

Schrodinger Cenatus: schrodinger.cenatus@umontreal.ca

Dan Zhang: 303102220034@stu.xzhmu.edu.cn

Siwei Chu: siwei.chu@umontreal.ca

Nathalie Henley : nhenley.hmr@ssss.gouv.qc.ca

Vincent Pichette: vincent.pichette@umontreal.ca

Jonatan Barrera-Chimal: barrera.chimal.jonatan.cemtl@ssss.gouv.qc.ca

Casimiro Gerarduzzi: casimiro.gerarduzzi@umontreal.ca

***Correspondence:** Dr. Casimiro Gerarduzzi

Maisonneuve-Rosemont Hospital Research Center, CIUSSS de l'Est-de-l'Île-de-Montréal 5345, boul. de l'Assomption, Montreal, Quebec, Canada H1T 2M4

Email: casimiro.gerarduzzi@umontreal.ca

Phone: (514) 252-3400 ext.2813

**Supplementary Materials and Methods**

**Chemicals and reagents**

Dimethylsulfoxide (DMSO, Catalog No. D8418), dimethylformamide (DMF, Catalog No. 22056), ammonium persulfate (APS), Ponceau S, tetramethylethylenediamine (TEMED), sodium citrate (SX0445-1) was purchased from Sigma-Aldrich. Acrylamide, ethylenediaminetetraacetic acid (EDTA), bis-acrylamide, sodium dodecyl sulfate (SDS), Tween 20, methanol, Tris-base, and glycine were purchased from VWR International, and 10% buffe. All chemicals were of ACS grade or higher.

**Serum creatinine and blood urea nitrogen measurements**

Mouse serum creatinine (Scr) levels were measured using the Mouse Creatinine Assay Kit (Crystal Chem, Catalog No. 80350) according to the manufacturer's instructions. This assay is based on a fully enzymatic method, which significantly reduces interference compared to the traditional alkaline picric acid method. Blood urea nitrogen (BUN) levels were quantified using the QuantiChrom Urea Assay Kit (BioAssay Systems, Catalog No. DIUR-100) following the manufacturer's instructions.

**Transcriptome analysis using RNA-seq**

Snap-frozen kidneys were crushed using polytron homogenizers in TRIzol (Invitrogen, Catalog No.15596026), and extracted RNA was cleaned up with Qiagen mRNA cleanup kit. RNA samples were submitted to Genome Québec for quality assessment, library preparation, and sequencing on the Illumina HiSeq X10 platform. Each sample yielded a minimum of 50 million reads. Sequencing data were aligned to the Mus musculus GRCm38_v100 genome assembly. The resulting FASTQ files were further processed and analyzed by the Bioinformatics Core Facility at the Institut de Recherches Cliniques de Montréal (IRCM) to generate transcriptomic profiles and downstream bioinformatics analyses.

**Gene set enrichment analysis (GSEA)**

GSEA analysis was performed using the GSEA software (version 4.3.3 Broad Institute) to evaluate the enrichment of specific biological processes in transcriptomic data. Custom gene sets representing cell proliferation, tubular injury, and DNA repair were curated based on published literature, with the full gene lists provided in Supplementary Table S2. The analysis was conducted in preranked mode using log2 fold-change values derived from differential gene expression between SMOC2 WT and KO AAN mice.

**Palbociclib treatment (In Vivo)**

Palbociclib (HY-50767, MedChemExpress) was dissolved in 10 mM sodium citrate buffer (pH 6.0) to a final concentration of 10 mg/mL. For preventive treatment, mice were treated with a 150 mg/kg BW dose of palbociclib, or vehicle, 4 hours before AAI injection by oral gavage (p.o.). The dose and time point selection are based on previous publications [1-3]. For therapeutic treatment, mice were treated with the same dose of palbociclib, or vehicle, 4hours or 24hours after AAI injection by oral gavage. All mice were sacrificed on day 4.

**Recombinant SMOC2 protein treatment (In Vivo)**

Human recombinant SMOC2 (rSMOC2) protein (R&D, 5140-SM) was reconstituted at 200 μg/mL in PBS. Mice were treated with a 200 ng/kg BW dose of rSMOC2, or PBS, 24 hours after AAI injection by i.p. for 3days, and mice were sacrificed on day 4.

**Histological staining**

Paraffin-embedded kidney tissue sections (5 μm) were stained to visualize the basic kidney structure using hematoxylin (Sigma, Catalog No. HT11026) and eosin (Sigma, Catalog No. GHS216) (HE) following a standard protocol. Masson's Trichrome Stain Kit (Abcam, Catalog No. ab150686) was used to detect collagen deposition in the kidney according to the manufacturer's instructions.

**Tubule injury score.**

Tubular injury was assessed based on HE staining. Tubular injury was defined as tubular dilation, tubular atrophy, tubular cast formation, vacuolization, degeneration, and sloughing off of tubular epithelial cells or loss of the brush border and thickening of the tubular basement membrane. 6 non-overlapping fields in renal cortex were scored (200× magnification) using the following tubular injury system as previously described [4]: 0 = no tubular injury; 1-10% tubules injured; 2 = 11%-25% tubules injured; 3 = 26%-50% tubules injured; 4 = 51%-74% tubules injured; and 5 = >75% tubules injured.

**Immunohistochemistry staining**

5μm thick paraffin-embedded kidney sections were deparaffinized and rehydrated. Antigen retrieval was performed using 10 mM citrate buffer (pH 6.0) with 0.05% Tween 20 at 95 °C for 10~15 min. After blocking with 5% donkey serum, the sections were incubated with the respective antibodies at the dilutions specified in Supplemental Tables S3 and S4. Fluoroshield with DAPI (Millipore-Sigma, F6057) was used for nuclear staining and mounting. Tile scan images were acquired with a Zeiss AxioObserver.Z1 inverted microscope at 10 × magnification. Nonoverlapping high power fields (HPF) (magnification 400x) were used for quantitative analysis, and the number of positive cells/tubules/mean fluorescence intensity (MFI) was determined as the average of positive signals of at least 6 fields per kidney section.

**Terminal deoxynucleotidyl transferase dUTP nick-end labeling (TUNEL) assays**

TUNEL assays were performed using the In Situ Cell Death Detection Kit, TMR Red (REF 12156792910, Roche) according to the manufacturer’s instructions. Briefly, 5-μm paraffin-embedded kidney sections were deparaffinized and rehydrated. The sections were then permeabilized with a freshly prepared buffer containing 0.1% Triton X-100 and 0.1% sodium citrate for 8 minutes at room temperature, followed by incubation with the TUNEL reagent mixture for 60 minutes at 37°C. After incubation, the sections were washed three times with PBS (5 minutes per wash) and mounted with an antifade mounting medium. Tile scan images were acquired using a Zeiss AxioObserver.Z1 inverted microscope at 10× magnification. TUNEL-positive cells were counted at 400× magnification in six fields per kidney and recorded.

**Immunocytochemistry staining**

HK-2 cells were seeded on 8-well removable chamber slide (ibidi, 80841) and fixed with 4% PFA, blocked with 5% BSA, and incubated with anti-ITGB3 (1:100; Cell signaling, #13166) and anti-Myc (1:400; Cell signaling, #2276) at 4°C overnight. After washing with PBS, they were incubated with secondary antibodies conjugated with Alexa Fluor 647 and Cy3. Cells were counterstained with DAPI to visualize the nuclei and examined by aforementioned microscopy. For primary mTECs purity verification, freshly isolated mTECs were seeded in 12-well plates and processed using the same staining protocol as for HK-2 cells. The primary antibodies used for purity identification are listed in Supplementary Table S3.

**Western blot analysis**

Cold radioimmunoprecipitation assay (RIPA) buffer (Thermo Fisher Scientific, Cat. No. 89901) containing phosphatase (Roche, Cat. No. 05892791) and protease inhibitors (Roche, Cat. No. 04906837) was used to homogenize kidney tissues and cell cultures. The concentration of cellular lysate was determined using a bicinchoninic acid (BCA) protein assay kit (Pierce, Cat. No. 23225). Protein lysates (20 μg for cellular lysate and 40 μg for kidney lysate) for separation by electrophoresis on 8~12% polyacrylamide gels containing 0.4% SDS, followed by transfer onto 0.22 μm polyvinylidene difluoride (PVDF) membranes (Bio-Rad). The membranes were incubated with the primary and secondary antibodies at the dilutions specified in Supplemental Tables S3 and S4. Bands were detected with the Clarity Max Western ECL Substrate (Bio-Rad). Results were analyzed using ImageJ.

**Quantitative real-time PCR**

RNA was extracted using TRIzol, and then purified using the RNeasy Mini kit (Qiagen) according to the manufacturer’s protocol. One microgram of total RNA was reverse transcribed into cDNA using SuperScript VILO cDNA Synthesis kit with ezDNase (Invitrogen). qPCR was performed using SsoAdvanced Universal SYBR Green Supermix (Bio-Rad) on an ABI 7500 Real-Time PCR System (Applied Biosystems). The list of primers can be found in Supplemental Table S1. All samples were measured with technical triplicates and normalized against average housing-keeping gene (HK) expression, including Gapdh, Hprt1, *Rplp0*. Primer amplification efficiencies were calculated for each gene and changes in the mRNA expression were determined using the Pfaffl method.

**Cell culture**

The human proximal tubule cell line (HK-2) was obtained from American Type Culture Collection (ATCC, USA) and maintained in a humidified atmosphere of 5% CO_2_ at 37°C with DMEM/F12 supplemented with heat-inactivated 10% FBS. Cell line authentication was performed using the GenePrint® 10 short tandem repeat (STR) profiling system. This analysis revealed an 83.3% similarity to the reference STR profile, confirming a single-source human male STR profile and excluding cross-contamination or misidentification. In addition, cells were confirmed to be free of mycoplasma contamination by a PCR-based assay [5].

**Cellular transfection and treatment with AAI, rSMOC2, and palbociclib**

HK-2 cells and mTECs were treated with aristolochic acid I (AAI, 20 μg/mL) for 72 hours to induce cellular apoptosis. For transfection experiments, HK-2 cells (1 × 10^5^) were seeded in 6-well plates and transfected with 100 pmol of scramble siRNA (Santa Cruz, sc-37007), SMOC2 siRNA (Santa Cruz, sc-63046), or ITGB3 siRNA (Santa Cruz, sc-29375), or 2 μg of Myc-SMOC2 plasmid using Lipofectamine 2000 (Invitrogen) followed by 20 μg/mL AAI treatment. For palbociclib treatment, a dose-response analysis (0, 1, 5, and 10 μM) was performed. Results indicated that pretreatment with 10 μM palbociclib for 3 hours effectively inhibited AAI-induced Rb phosphorylation in HK-2 cells. Similarly, a titration of rSMOC2 (0, 1, 10, 100 ng/mL) was conducted, revealing that pretreatment with 1 ng/mL rSMOC2 for 5 hours significantly attenuated AAI-induced DNA damage and apoptosis in HK-2 cells.

**Cell cycle analysis**

HK-2 cells subjected to different treatments were trypsinized and prepared single cell suspension at ~1 x 10^6^ cells/mL in PBS buffer. Aliquot 200 µl cells (~2 x 10^5^) in a 1.5 ml Eppendorf tube and add 500 µl cold 100% ethanol dropwise and gently mix by pipetting in and out at least ten times (ethanol final concentration will be ~70%) and fixed at 4°C for 1 hr. After being washed with PBS, the cells were incubated out of light in PBS containing 50 μg/mL propidium iodide (VWR, item No. 89139-066) and 10 μg/mL RNaseA (Sigma, R4875,) at 4°C for 2.5 hrs. Then, before measurement, the cell samples were resuspended in PBS and analyzed with a FACS Calibur flow cytometry (Becton Dickinson, USA).

**Co-Immunoprecipitation (Co-IP) analysis**

HK-2 cells were transfected with 8 μg of Myc-SMOC2 or Myc-empty vector per 10 cm dish using Lipofectamine 2000. For co-transfection experiments, a total of 10 μg of plasmid DNA was used at a 1:1 ratio of Myc-ITGB3 to truncated SMOC2 constructs. Forty-eight hours after transfection, cells were lysed in IPH buffer (50 mM Tris-HCl, pH 8.0; 150 mM NaCl; 5 mM EDTA; 0.5% NP-40) supplemented with a 1× protease and phosphatase inhibitor cocktail. Protein concentrations were determined using the BCA assay. For immunoprecipitation (IP), approximately 1 mg of total protein was incubated overnight at 4 °C with either anti-Myc agarose beads (Sigma-Aldrich, Cat. #A7470) or Protein G agarose beads (Sigma-Aldrich, Cat. #11719416001) together with an ITGB3 antibody (12 µL/IP, CST #13166). The beads were collected by centrifugation and washed three times with IPH buffer to remove non-specifically bound proteins. To avoid interference from immunoglobulin heavy and light chains during detection of truncated SMOC2, Myc-bead-bound proteins were eluted with 50 µL of c-Myc peptide (GenScript, Cat. #RP11731-5) reconstituted in PBS at 1 mg/mL, followed by incubation at 37 °C with shaking for 10 minutes. The supernatant was collected, mixed with a 5× sample buffer, and boiled for 5 minutes before Western blot analysis. For standard IP elution, bead-bound complexes were boiled directly in a 2× sample buffer for 5 minutes. The resulting supernatants were analyzed by Western blotting.

**Human kidney biopsy**

Human kidney sections were obtained from the Department of Nephrology, the Affiliated Hospital of Xuzhou Medical University. Two renal cortical samples adjacent to renal cell carcinoma (RCC) were used as healthy controls, and three kidney biopsy samples were collected from patients with AKI superimposed on CKD.

**Mycoplasma detection by PCR**

Mycoplasma contamination was assessed by a PCR-based assay based on a previously published method [5], targeting conserved regions of the mycoplasma 16S rRNA gene using a set of broad-spectrum primers. Each PCR run included a plasmid-based internal control (1000bp) consisting of a pGEM plasmid containing the 16S rRNA PCR product of Acholeplasma laidlawii, a DNA preparation from mycoplasma-infected cell culture supernatant as an additional positive control (500bp), and a no-template negative control. PCR products were analyzed by 1.5% agarose gel electrophoresis, and the presence of a band of the expected size was considered indicative of mycoplasma contamination. Randomly sampled culture supernatants from HK-2 cells tested negative for mycoplasma contamination.

**Supplementary Figures**

**
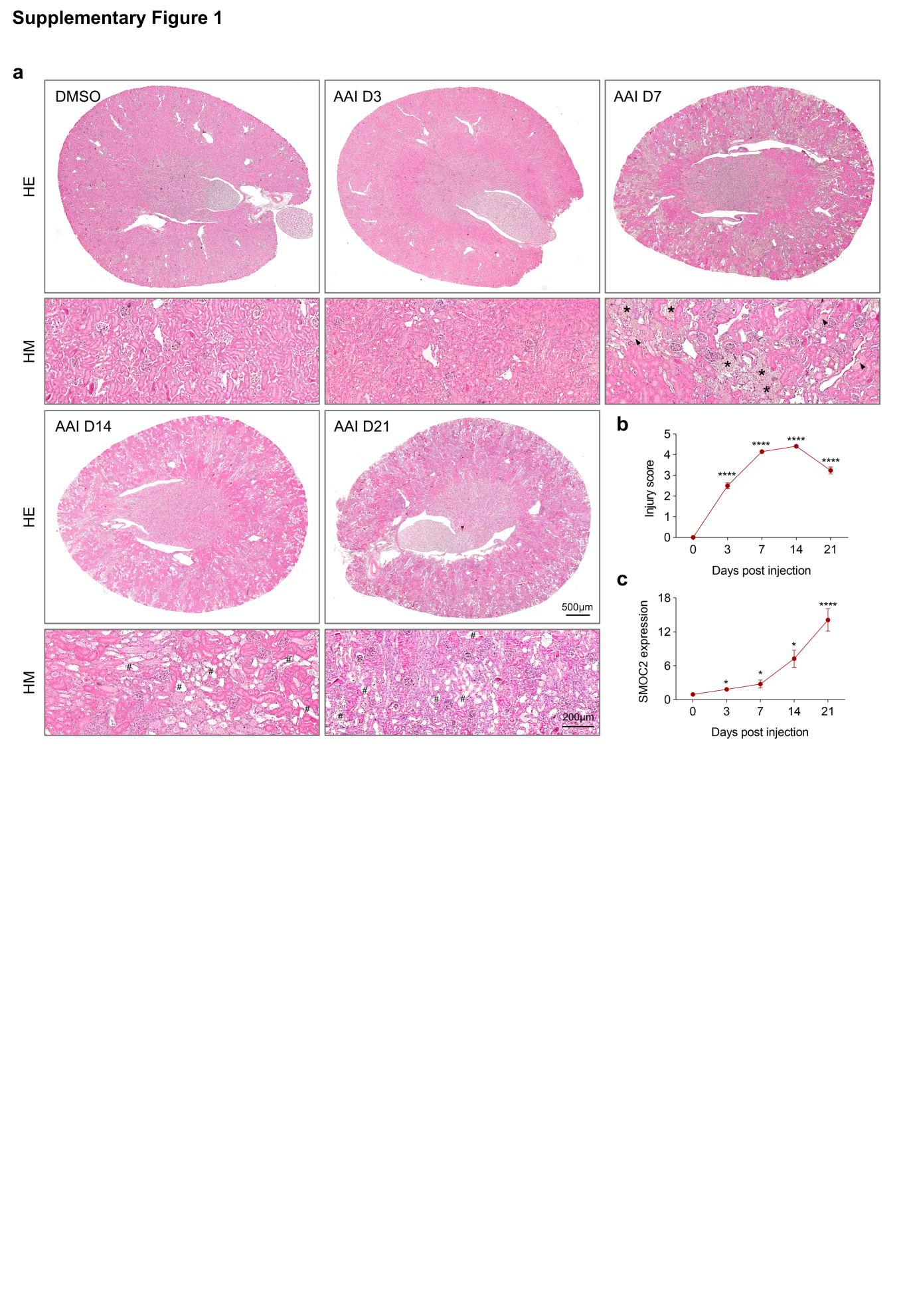
**

**Supplementary Fig. 1 Histopathological changes in the kidney during the AAI-induced AKI-to-CKD transition in male mice.**

**a** Representative HE-stained kidney sections from male WT mice treated with AAI (5 mg/kg body weight, BW) and collected at days 3, 7, 14, and 21 after injection. Asterisks indicate tubular lysis, arrowheads denote tubular casts, and pound signs (#) mark tubular atrophy.

**b** Changes in the kidney injury score during the course of AAI-induced AKI-to-CKD transition.

**c** Quantification of SMOC2 protein expression corresponding to Figure 1c at days 3, 7, 14, and 21 post-AAI injection.

n = 6 per time point, p*<0.05, p****<0.0001. vs. DMSO group

**
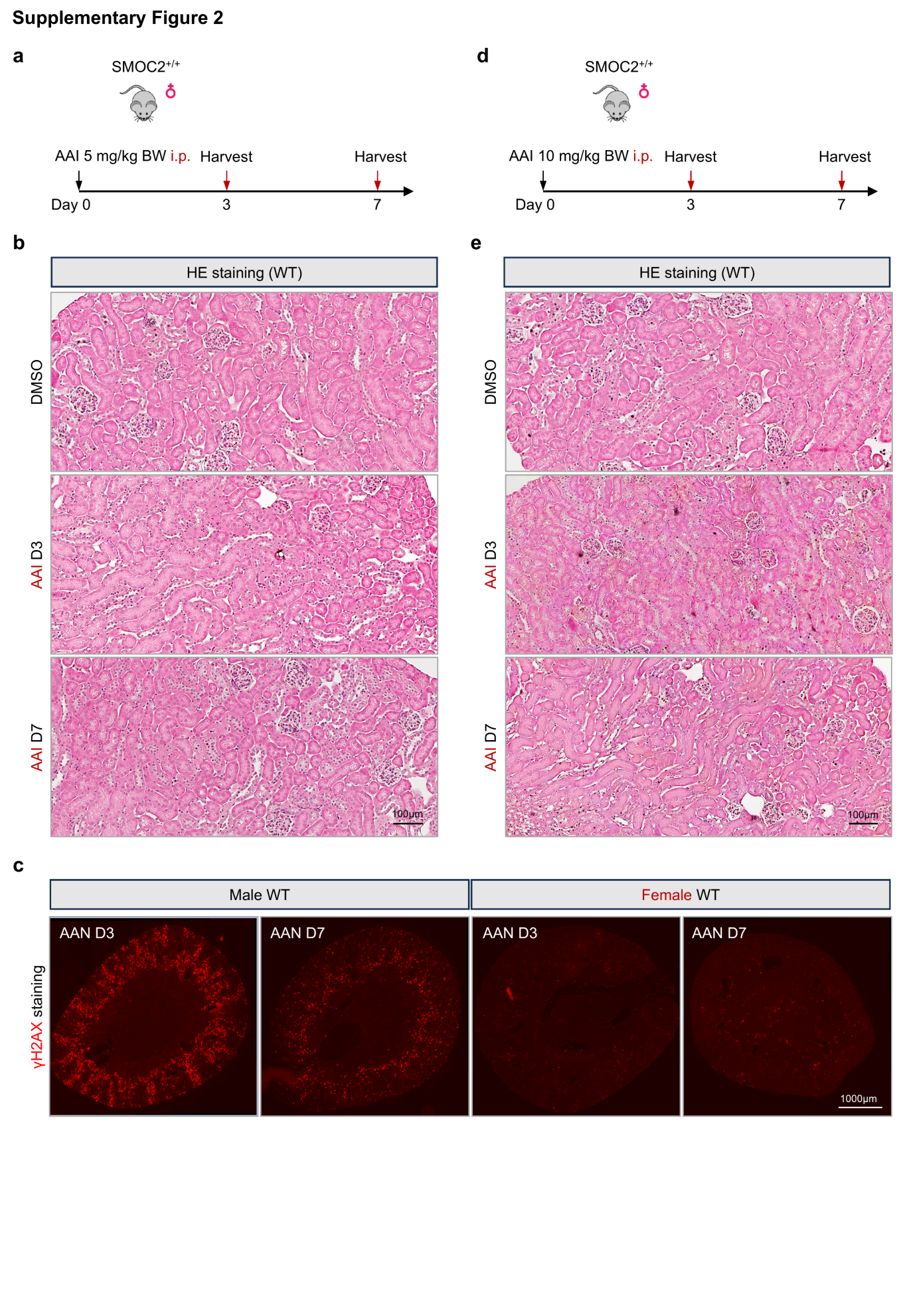
**

**Supplementary Fig. 2** **Female mice exhibit resistance to AAI-induced kidney injury following a single AAI injection (5 mg/kg or 10 mg/kg BW).**

**a** Schematic diagram illustrating the 5 mg/kg BW AAI-induced AKI protocol in female WT mice.

**b** Representative HE-stained kidney sections from female WT mice treated with AAI, collected at day 3 and day 7.

**c** Representative immunofluorescence staining for γH2AX in kidney sections from AAI-treated male and female WT mice at day 3 and day 7, showing differences in DNA damage response.

**d** Schematic diagram illustrating the 10 mg/kg BW AAI-induced AKI protocol in female WT mice.

**e** Representative HE-stained kidney sections from female mice treated with 10 mg/kg BW AAI, collected at day 3 and day 7.

n = 3-6 per time point.


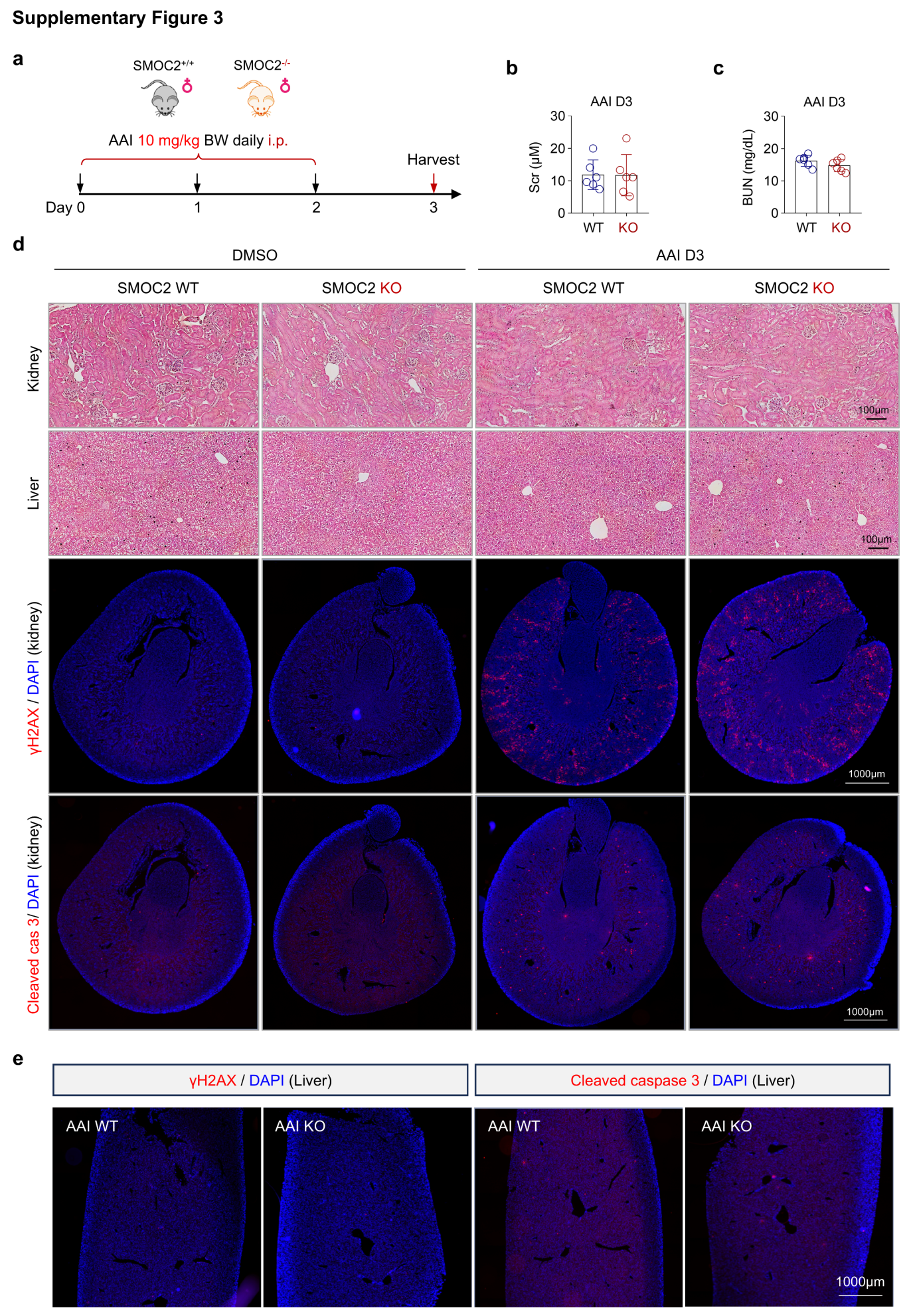


**Supplementary Fig. 3** **Female mice exhibit minimal response to AAI-induced kidney injury following daily AAI injection (10 mg/kg BW).**

**a** Schematic diagram illustrating the 10 mg/kg BW AAI daily injection protocol for inducing AKI in female SMOC2 WT and KO mice.

**b** Scr levels in female SMOC2 WT and KO AAN mice at day 3.

**c** BUN levels in female SMOC2 WT and KO AAN mice at day 3.

**d** First row: Representative HE-stained kidney sections from female SMOC2 WT and KO mice receiving daily AAI injections.

Second row: Representative HE-stained liver sections from female SMOC2 WT and KO mice following daily AAI injection, assessing potential toxicity in organs beyond the kidney.

Third row: Representative immunofluorescence staining for γH2AX in kidney sections from female SMOC2 WT and KO mice after daily AAI injection.

Forth row: Representative TUNEL staining in kidney sections from female SMOC2 WT and KO mice receiving daily AAI injections.

**e** Left panel: Representative immunofluorescence staining for γH2AX in liver sections from female SMOC2 WT and KO mice after daily AAI injection. Right panel: Representative TUNEL staining in liver sections from female SMOC2 WT and KO mice following daily AAI injection.

n =6 per AAN group.


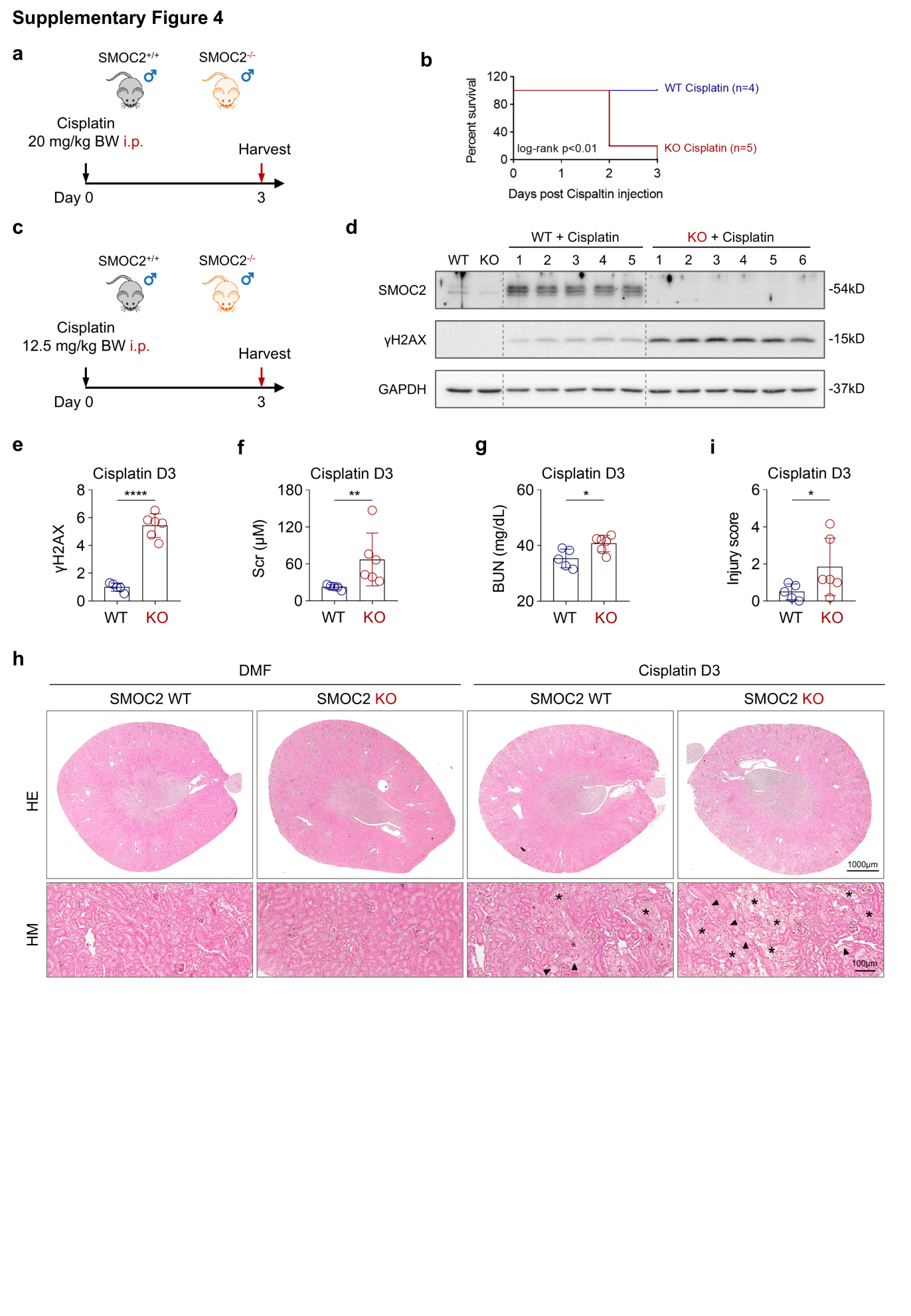


**Supplementary Fig. 4 SMOC2 knockout exacerbates cisplatin-induced acute tubular injury in male mice.**

**a** Schematic diagram illustrating the 20 mg/kg BW cisplatin injection protocol for inducing AKI in male SMOC2 WT and KO mice.

**b** Survival analysis of WT and SMOC2 KO mice following cisplatin injection (n = 4 for WT, n = 5 for KO mice).

**c** Schematic diagram illustrating the 12.5 mg/kg BW cisplatin injection protocol for inducing AKI in male mice (n = 5 for WT, n = 6 for KO mice).

**d** Western blot analysis of SMOC2 and γH2AX expression in kidney tissues from SMOC2 WT and KO mice following either DMF or cisplatin injection at day 3.

**e** Quantification of γH2AX expression corresponding to panel (d).

**f** Scr levels in male SMOC2 WT and KO mice at day 3 following cisplatin injection.

**g** BUN levels in male SMOC2 WT and KO mice at day 3 following cisplatin injection.

**h** Representative HE-stained kidney sections from SMOC2 WT and KO mice following either DMF or cisplatin injection. Asterisks indicate injured tubules; arrowheads indicate proteinaceous casts.

**i** Quantification of tubular injury scores in SMOC2 WT and KO mice treated with cisplatin.

p* < 0.05, p** < 0.01, p*** < 0.001, p**** < 0.0001

**
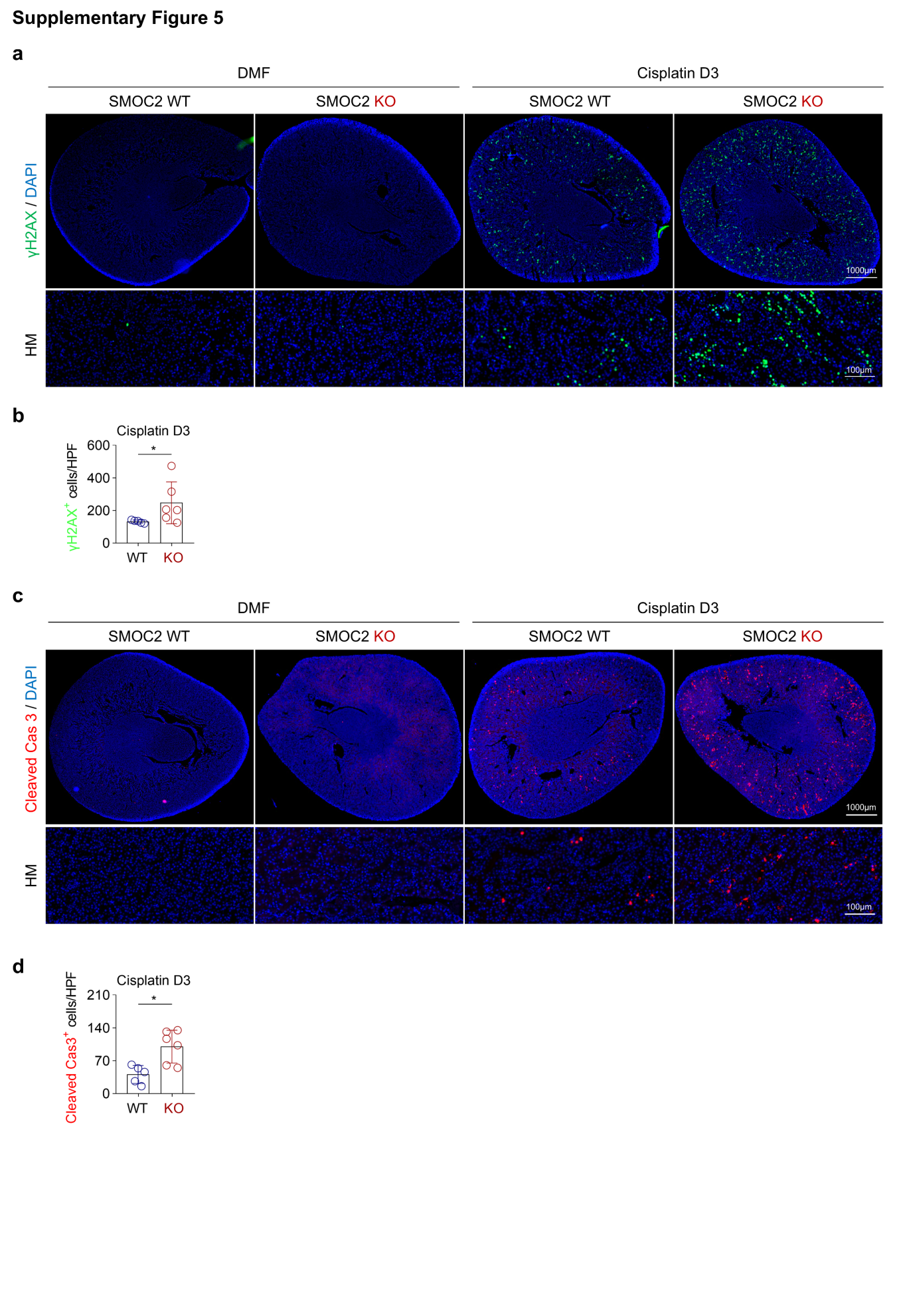
**

**Supplementary Fig. 5 SMOC2 knockout exacerbates cisplatin-induced DNA damage and cellular apoptosis in male mice.**

**a** Representative immunofluorescence staining for γH2AX in kidney sections from SMOC2 WT and KO mice following either DMF or cisplatin injection.

**b** Quantification of γH2AX^+^ cells per HPF in cisplatin-injected SMOC2 WT and KO mice at day 3.

**c** Representative cleaved caspase 3 staining in kidney sections from SMOC2 WT and KO mice following either DMF or cisplatin injection.

**d** Quantification of cleaved caspase 3^+^ per HPF in cisplatin-injected SMOC2 WT and KO mice at day 3.

n = 5 for WT + cisplatin, n = 6 for KO + cisplatin mice, p* < 0.05.

**
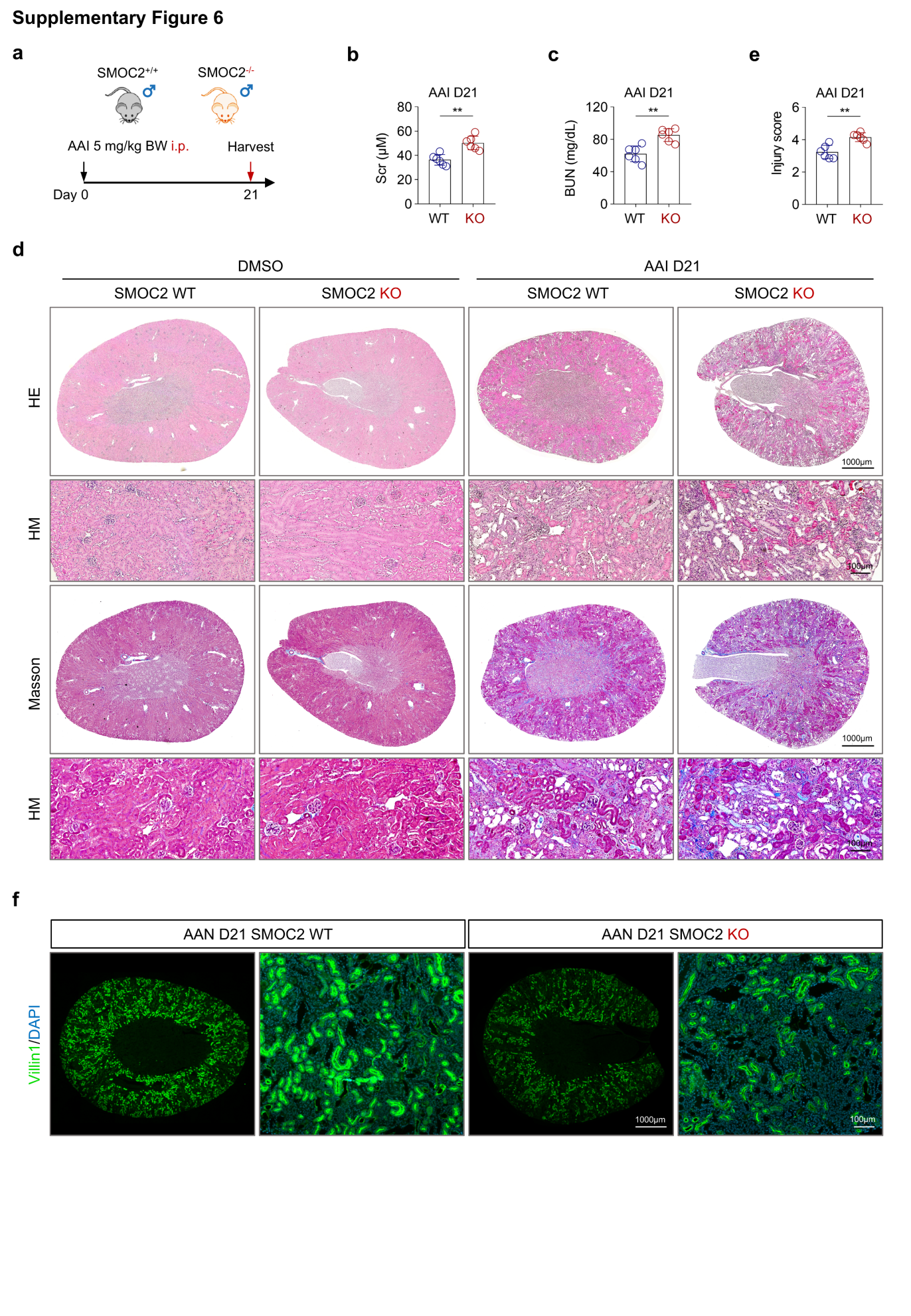
**

**Supplementary Fig. 6 SMOC2 knockout protects the kidney against acute tubular injury and subsequent renal fibrosis.**

**a** Schematic diagram illustrating the 5 mg/kg BW AAI injection protocol used to induce the AKI-to-CKD transition model in male SMOC2 WT and KO mice.

**b** Scr levels in SMOC2 WT and KO AAN mice at day 21 post-AAI injection.

**c** BUN levels in SMOC2 WT and KO AAN mice at day 21 post-AAI injection.

**d** Representative HE and Masson’s trichrome-stained kidney sections from SMOC2 WT and KO mice following either DMSO or AAI injection at day 21. Whole-slide scanned kidney images provide an overview of kidney injury and fibrosis. High-magnification HE images emphasize tubulointerstitial injury, while high-magnification Masson’s trichrome images distinctly highlight renal fibrosis.

**e** Quantification of tubulointerstitial injury scores in SMOC2 WT and KO AAN mice at day 21.

**f** Representative immunofluorescence staining for Villin1 in kidney sections from SMOC2 WT and KO mice at day 21 post-AAI injection, with high-magnification images demonstrating the preservation of tubular epithelial integrity.

n = 6 per group, p** < 0.01.


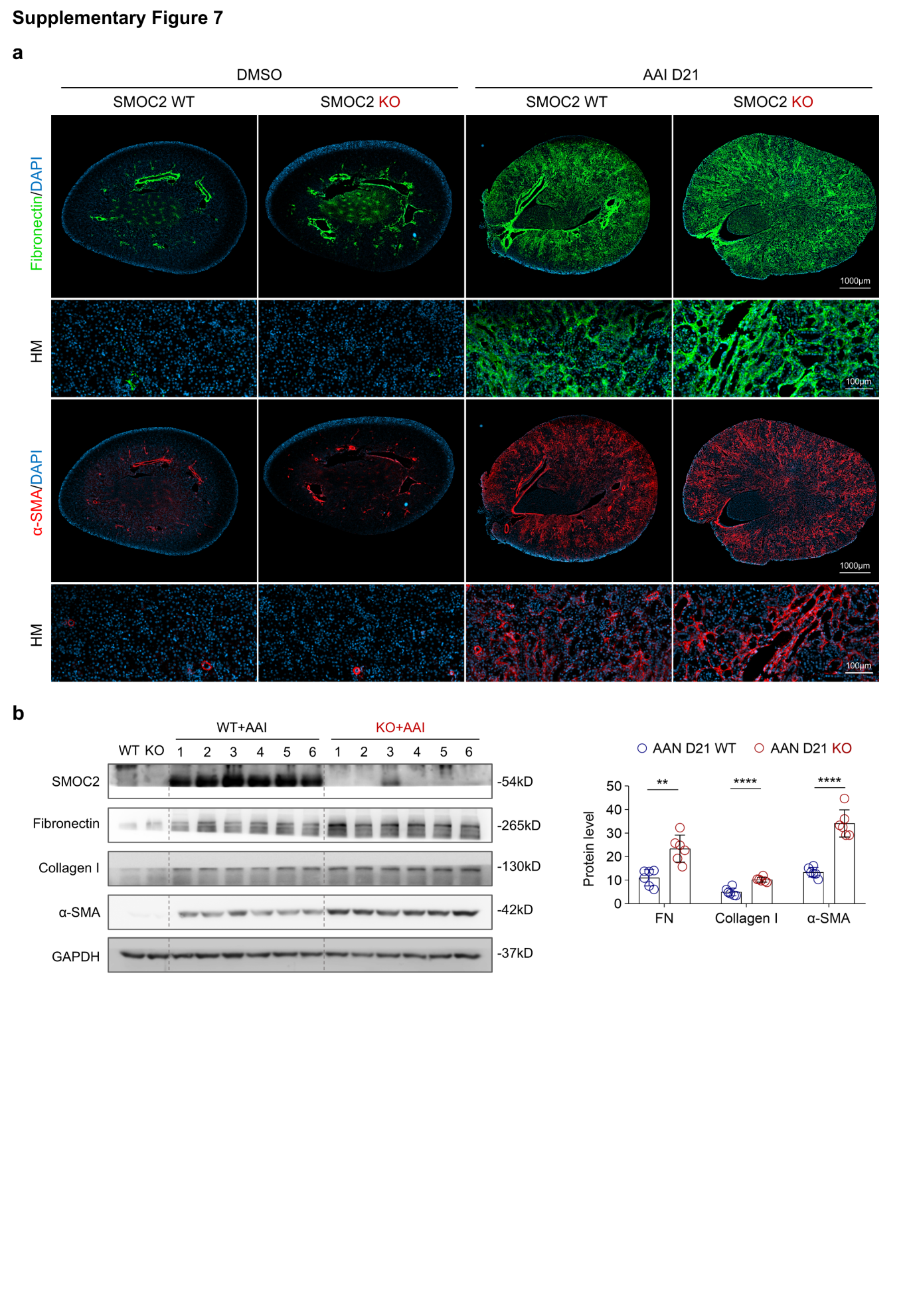


**Supplementary Fig. 7 SMOC2 knockout protects against AKI-induced renal fibrosis.**

**a** Representative immunofluorescence staining for fibronectin and α-SMA in kidney sections from SMOC2 WT and KO mice at day 21 post-AAI injection, with high-magnification images providing a detailed view of the fibrotic regions.

**b** Western blot analysis and quantification of SMOC2, fibronectin, collagen I, and α-SMA expression in kidney tissues from SMOC2 WT and KO mice following either DMSO or AAI injection at day 21.

n=6 per group, p** < 0.01, p**** < 0.0001.

**
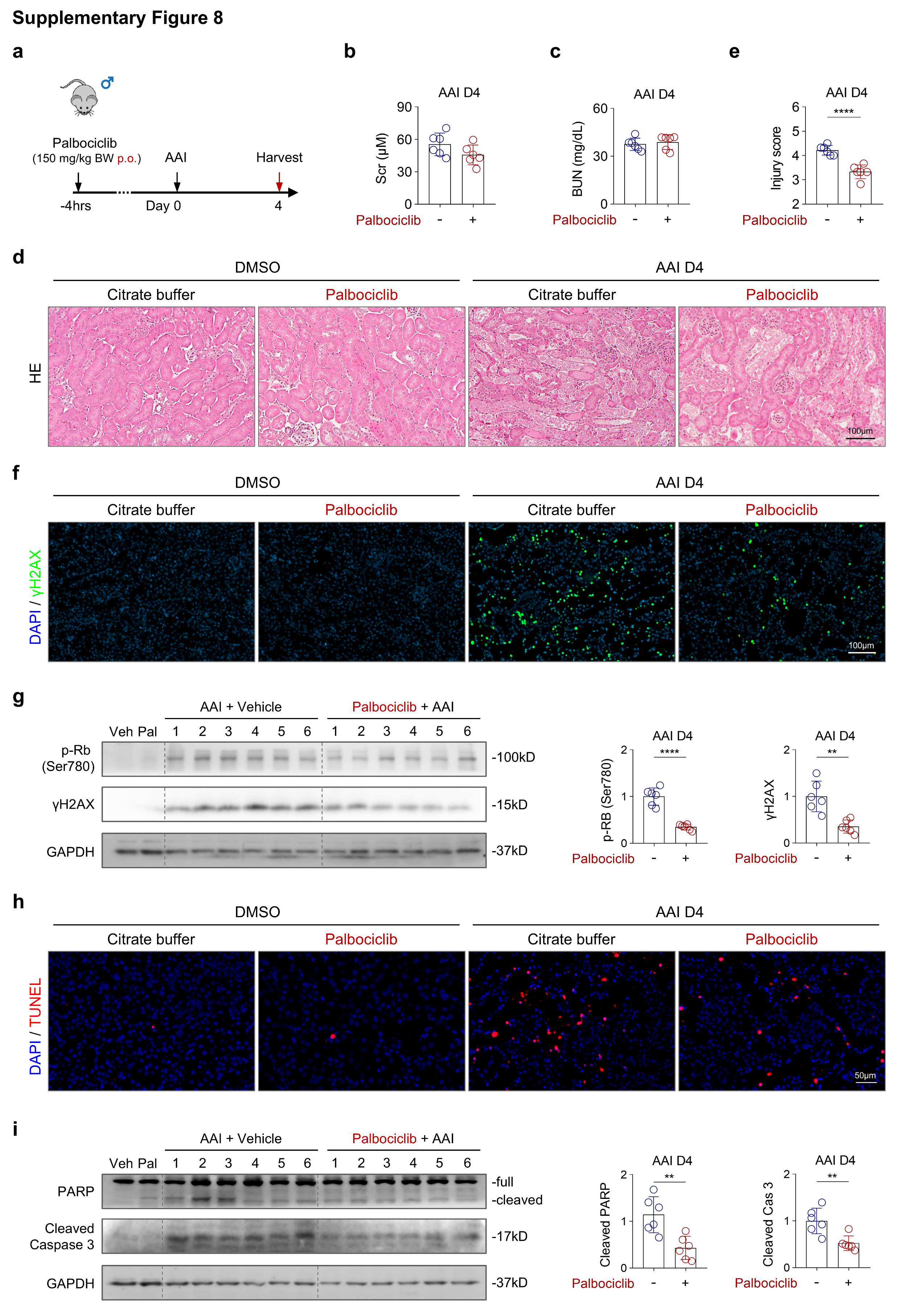
**

**Supplementary Fig. 8 Pre-injury treatment with palbociclib reduces DNA damage, apoptosis, and tubular injury in male AAN mice.**

**a** Schematic diagram illustrating the pre-injury treatment protocol with palbociclib in male AAN mice.

**b** Scr levels in AAN mice treated with palbociclib or citrate buffer.

**c** BUN levels in AAN mice treated with palbociclib or citrate buffer.

**d** Representative HE-stained kidney sections from DMSO- or AAI-treated mice following treatment with either palbociclib or citrate buffer.

**e** Quantification of tubular injury scores in AAN mice treated with palbociclib or citrate buffer.

**f** Representative γH2AX staining in kidney sections from DMSO- or AAI-treated mice following treatment with either palbociclib or citrate buffer.

**g** Western blot and quantification analysis of p-Rb (Ser 780) and γH2AX expression in kidney tissues from DMSO- or AAI-treated mice following treatment with either palbociclib or citrate buffer.

**h** Representative TUNEL staining in kidney sections from DMSO- or AAI-treated mice following treatment with either palbociclib or citrate buffer.

**i** Western blot and quantification analysis of PARP and cleaved caspase-3 expression in kidney tissues from DMSO- or AAI-treated mice following treatment with either palbociclib or citrate buffer.

n = 4 for the DMSO + citrate group, n = 5 for the DMSO + palbociclib group, and n = 6 for the AAN + citrate and AAN + palbociclib groups. p* < 0.05, p** < 0.01, p*** < 0.001, p**** < 0.0001.

**
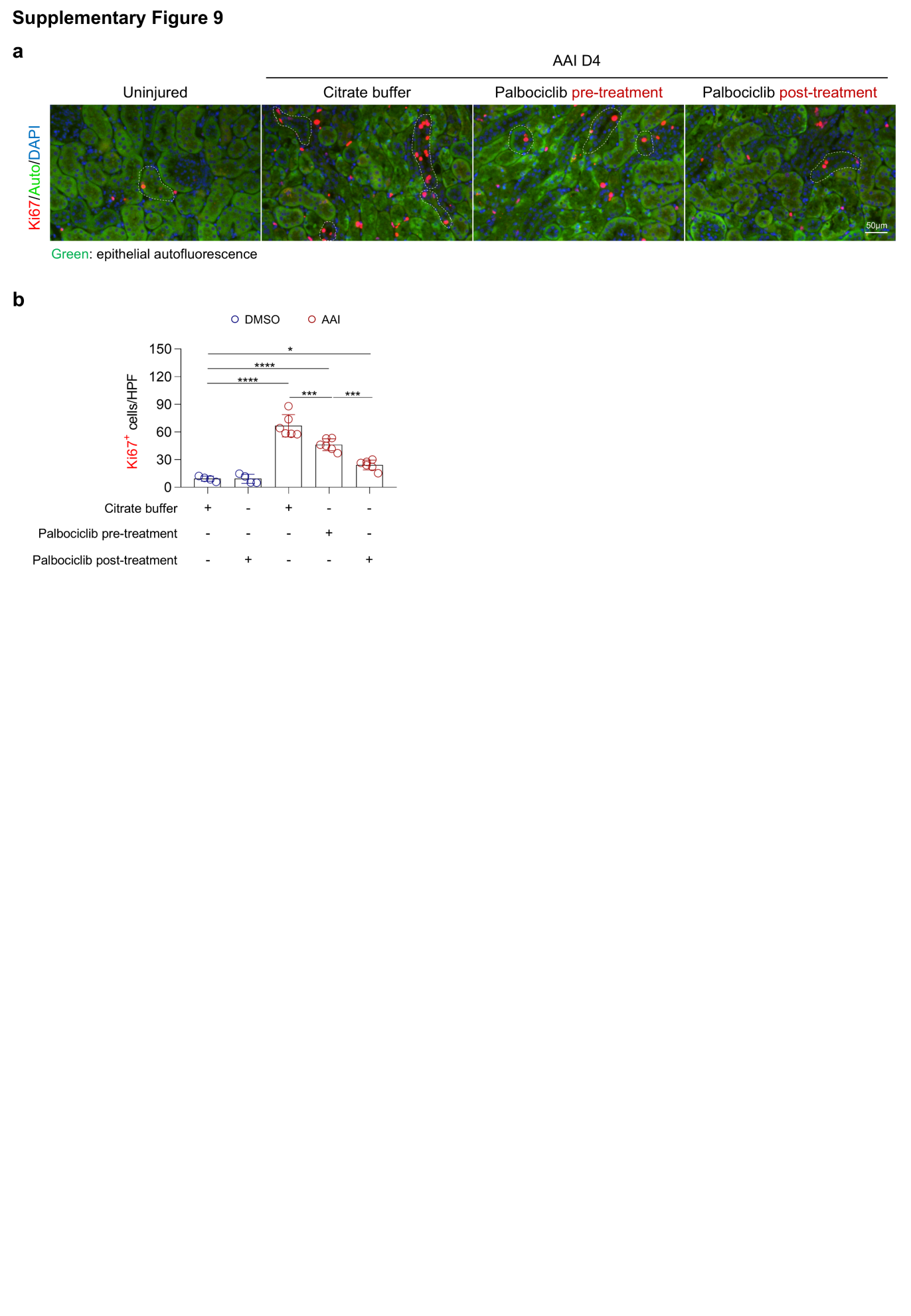
**

**Supplementary Fig. 9 Palbociclib treatment, administered either before or after AAI injection, effectively inhibits tubular proliferation.**

**a** Representative immunofluorescence staining for Ki67 (red), a marker of proliferation, in kidney sections from uninjured mice and AAN mice treated with either palbociclib or citrate buffer, demonstrating the impact of palbociclib on tubular cell proliferation. Green indicates intrinsic tubular epithelial autofluorescence visualized in the 488 nm channel, which delineates tubular structures.

**b** Quantification of Ki-67^+^ cells per HPF.

n = 4 for the DMSO + citrate group, n = 5 for the DMSO + palbociclib group, and n = 6 for the AAN + citrate, palbociclib+ AAN (pre-treatment), AAN + palbociclib groups (post-treatment). p* < 0.05, p** < 0.01, p*** < 0.001, p**** < 0.0001.

**
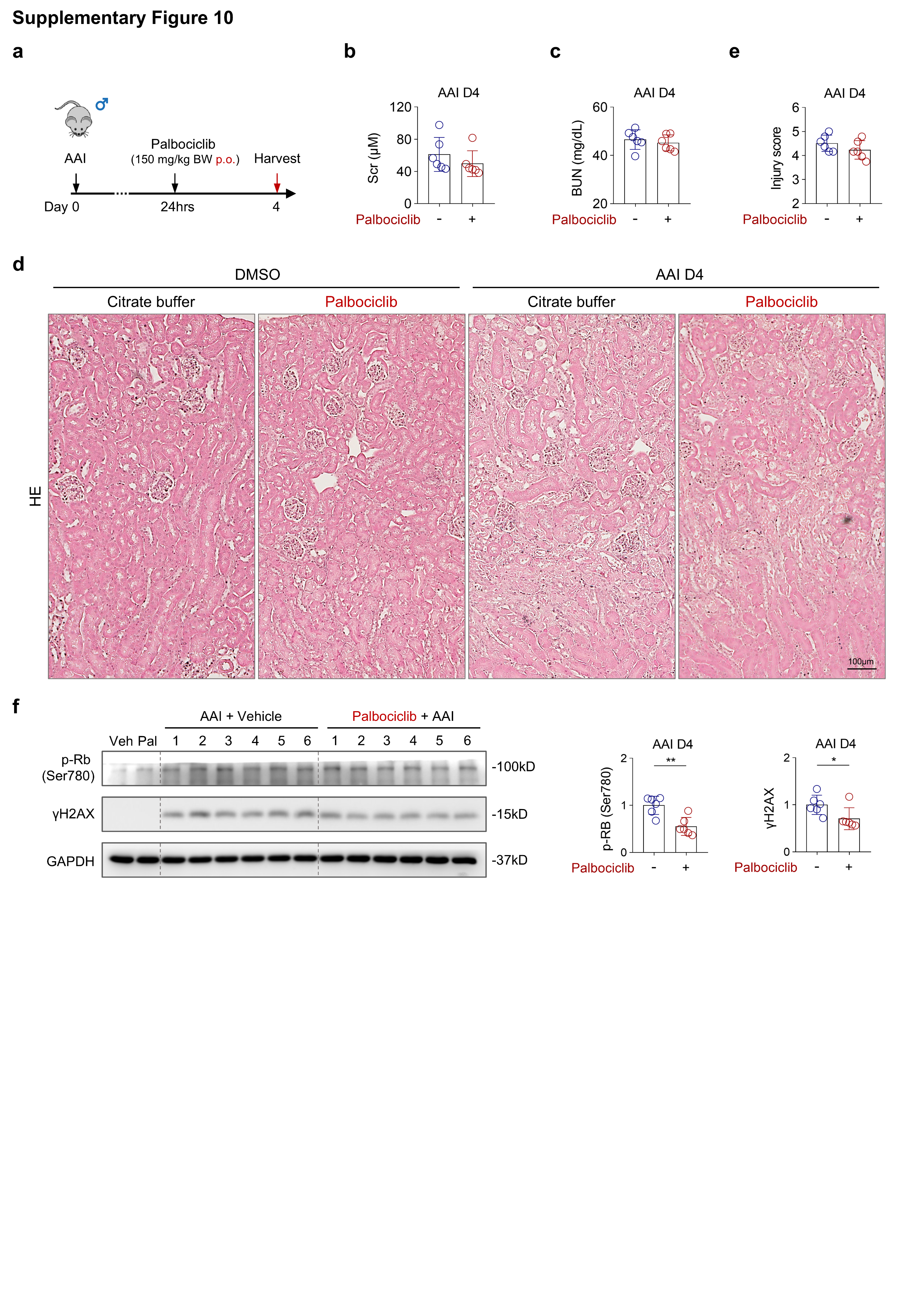
**

**Supplementary Fig. 10 Post-injury (24 h) treatment with palbociclib reduces DNA damage and shows a trend toward improved renal function and attenuated tubular injury in male AAN mice.**

**a** Schematic diagram illustrating the treatment protocol in which palbociclib was administered 24 hours after AAI-induced injury in male mice.

**b** Scr levels in AAN mice treated with palbociclib or citrate buffer.

**c** BUN levels in AAN mice treated with palbociclib or citrate buffer.

**d** Representative HE-stained kidney sections from DMSO- or AAI-treated mice following treatment with either palbociclib or citrate buffer.

**e** Quantification of tubular injury scores in AAN mice treated with palbociclib or citrate buffer.

**f** Western blot and quantification analysis of p-Rb (Ser 780) and γH2AX expression in kidney tissues from DMSO- or AAI-treated mice following treatment with either palbociclib or citrate buffer.

n = 4 for the DMSO + citrate group, n = 4 for the DMSO + palbociclib group, and n = 6 for the AAN + citrate and AAN + palbociclib groups. p* < 0.05, p** < 0.01, p*** < 0.001, p**** < 0.0001.

**
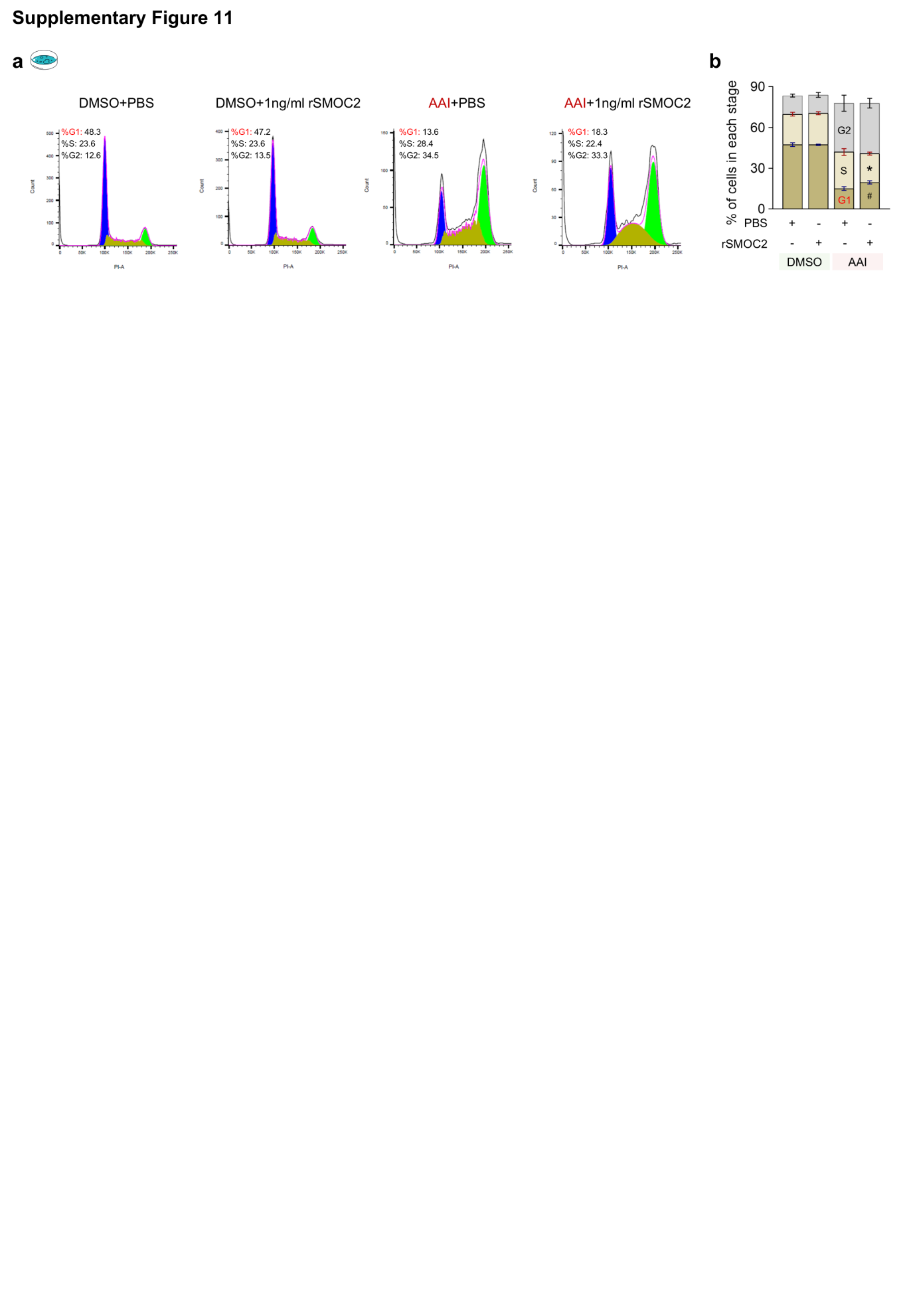
**

**Supplementary Fig. 11** **rSMOC2 treatment** **attenuates G1/S cell cycle transition in AAI-treated HK-2 cells.**

**a** Representative PI staining for cell cycle analysis in DMSO- and AAI-treated HK-2 cells, with or without 1 ng/ml rSMOC2, demonstrating the effect of rSMOC2 on G1/S phase progression.

**b** Quantification of cell cycle distribution in G1, S, and G2 phase.

n = 3 for each group, p^#^ < 0.05 vs AAI group cells in G1 cells, p* < 0.05 vs AAI group cells in S phase.


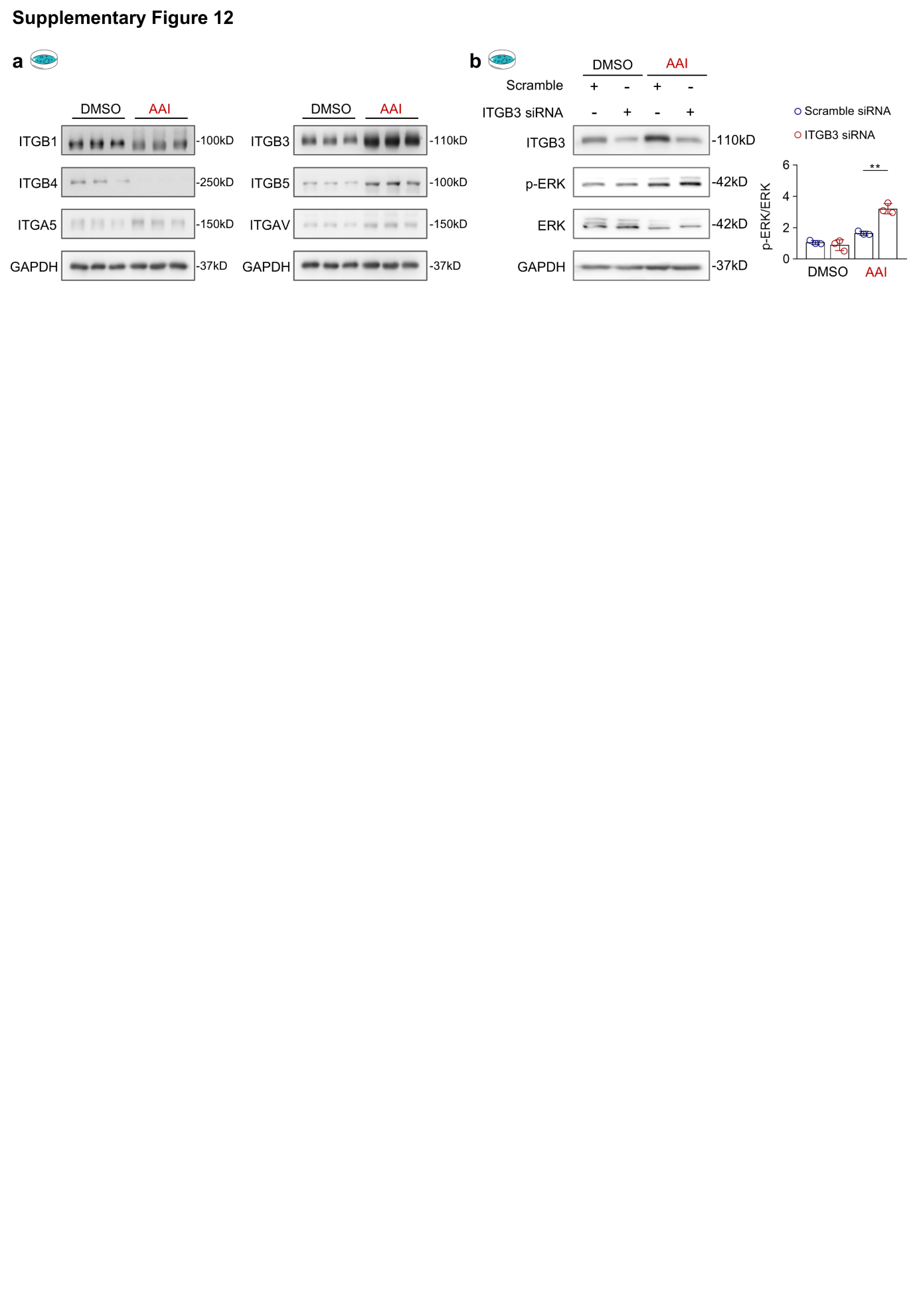


**Supplementary Fig. 12** **Analysis of integrin expression and the impact of ITGB3 knockdown on ERK activation in HK-2 cells following AAI treatment.**

**a** Western blot analysis of integrins expression in HK-2 cells treated with either DMSO or 20 μg/mL AAI.

**b** Western blot analysis of ITGB3, p-ERK, and total ERK expression in HK-2 cells transfected with either scramble siRNA or ITGB3 siRNA following treatment with 20 μg/mL AAI.

n = 3 per group. p**<0.01.


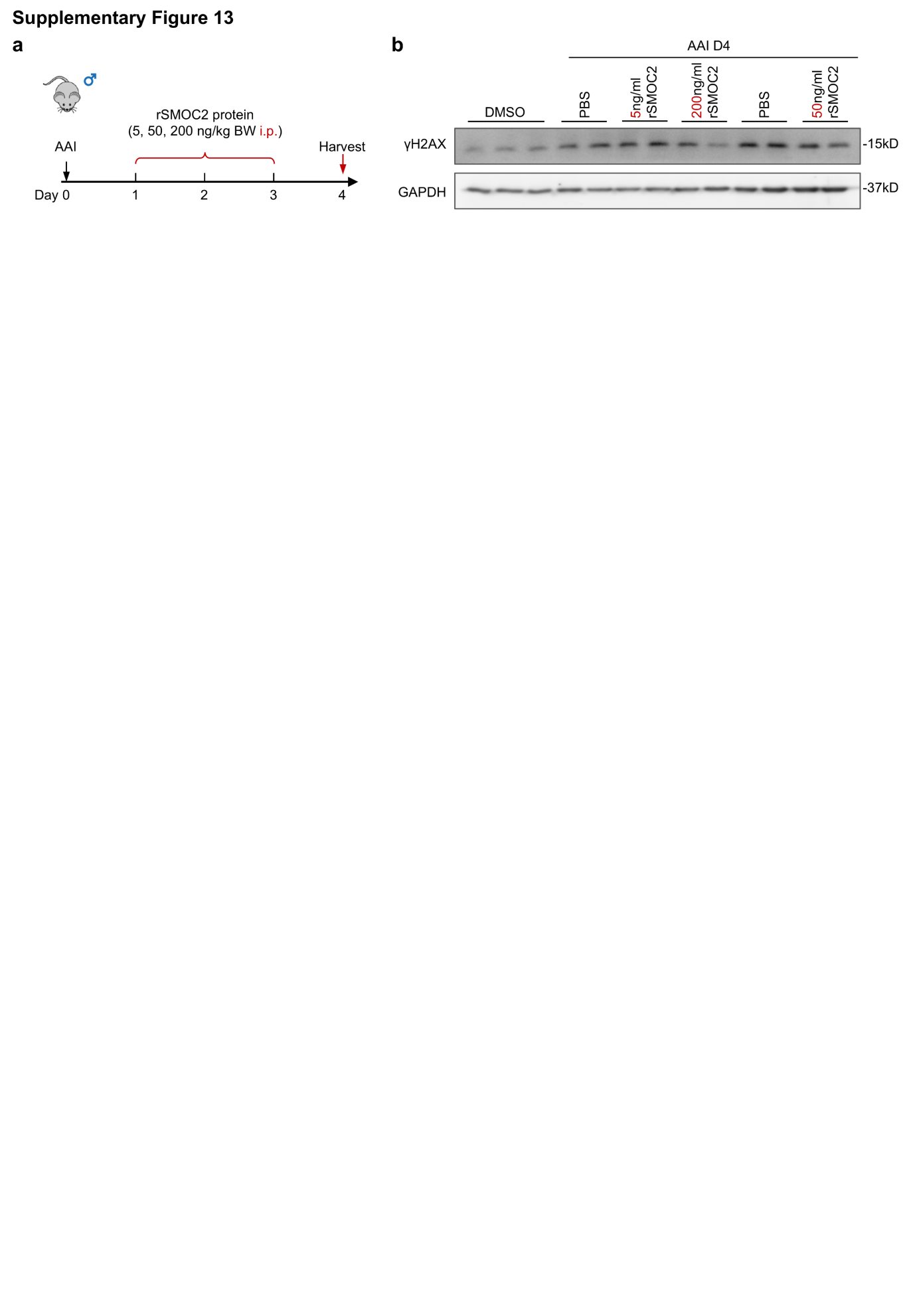


**Supplementary Fig. 13 Dose titration of rSMOC2 for the treatment of AAI-induced DNA damage.**

**a** Schematic illustration of the post-injury treatment protocol using different doses of recombinant SMOC2 (rSMOC2) in male AAN mice.

**b** Western blot analysis of γH2AX expression in kidney tissues from DMSO- or AAI-treated mice following intraperitoneal administration of rSMOC2 at 5, 50, or 200 ng/kg BW. n = 3 for the DMSO + PBS and AAI + PBS groups; n = 2 for each dose in the AAI + rSMOC2 groups.

**
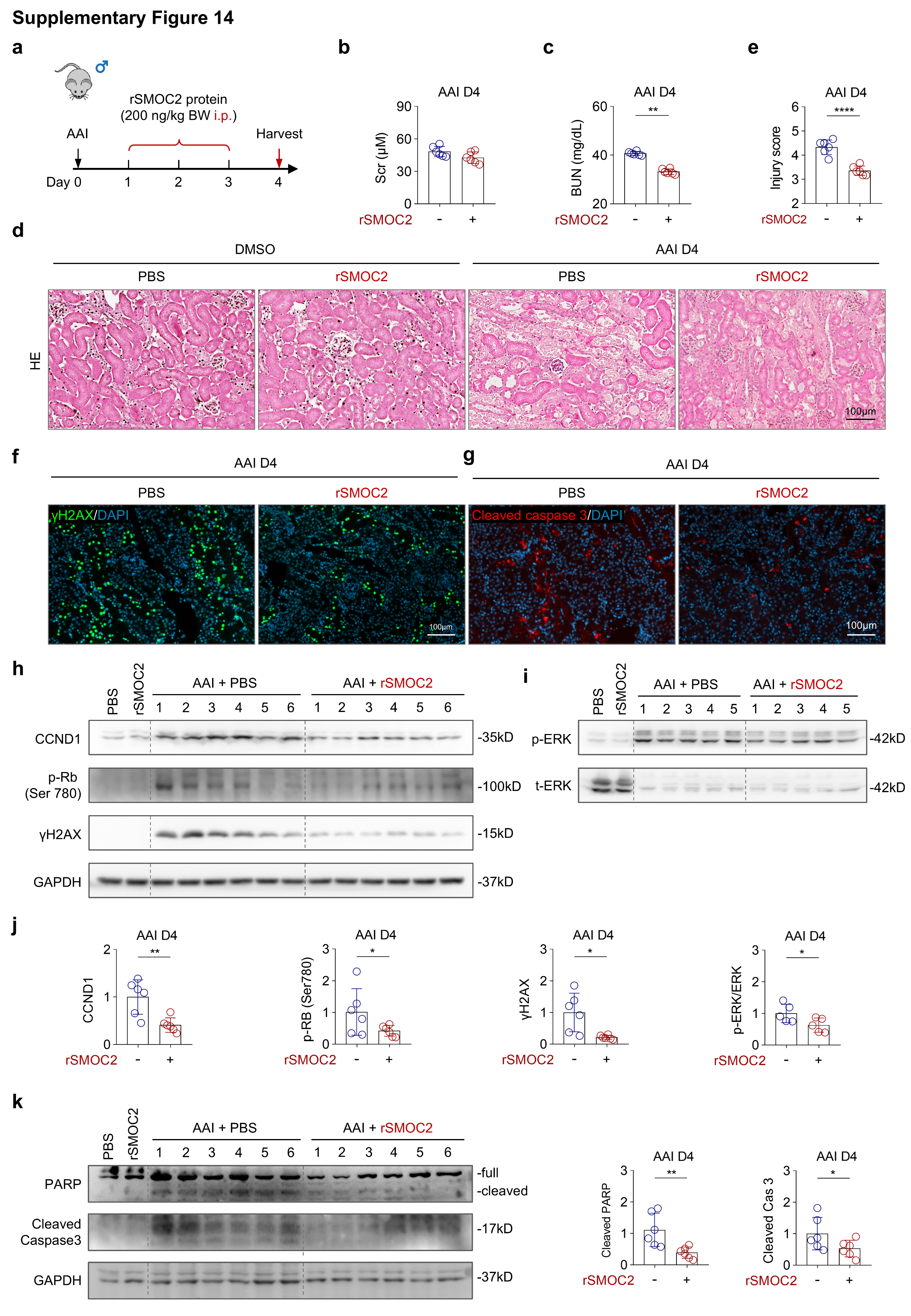
**

**Supplementary Fig. 14** **rSMOC2 treatment improves DNA damage, apoptosis, and tubular injury in male AAN mice.**

**a** Schematic diagram illustrating the post-injury treatment protocol with rSMOC2 in male AAN mice.

**b** Scr levels in AAN mice treated with rSMOC2 or vehicle (PBS).

**c** BUN levels in AAN mice treated with rSMOC2 or PBS.

**d** Representative HE-stained kidney sections from DMSO- or AAI-treated mice following treatment with either rSMOC2 or PBS.

**e** Quantification of tubular injury scores in AAN mice treated with rSMOC2 or PBS.

**f** Representative γH2AX staining in kidney sections from AAN mice following treatment with either rSMOC2 or PBS.

**g** Representative TUNEL staining in kidney sections from AAN mice following treatment with either rSMOC2 or PBS.

**h** Western blot analysis of CCND1, p-Rb Ser 780 and γH2AX expression in kidney tissues from DMSO- or AAI-treated mice following treatment with either rSMOC2 or PBS.

**i** Western blot analysis of p-ERK and total ERK expression in kidney tissues from DMSO- or AAI-treated mice following treatment with either rSMOC2 or PBS.

**j** Quantification analysis of CCND1, p-Rb Ser 780, γH2AX expression, and p-ERK/ERK ratio in kidney tissues from AAN mice following treatment with either rSMOC2 or PBS.

**k** Western blot and quantification analysis of PARP and cleaved caspase 3 expression in kidney tissues from DMSO- or AAI-treated mice following treatment with either rSMOC2 or PBS. n = 6 for the AAN + PBS and AAN + rSMOC2 groups. p*<0.05, p**<0.01, p***<0.001, p****<0.0001.

**
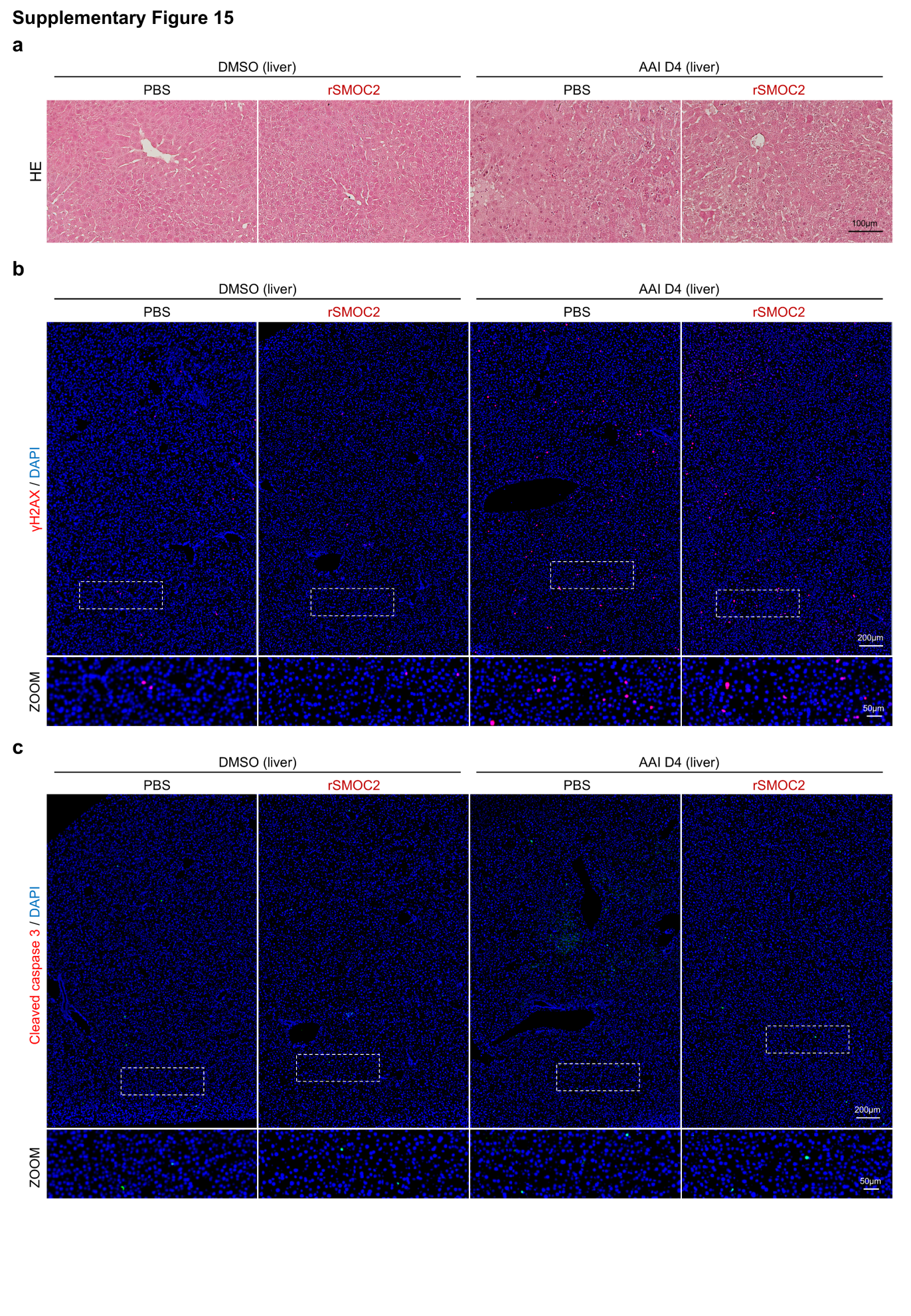
**

**Supplementary Fig. 15 Evaluation of potential hepatic side effects of rSMOC2 treatment in AAN mice.**

**a** Representative HE-stained liver sections from DMSO- or AAI-treated mice following treatment with either rSMOC2 or PBS.

**b** Representative immunofluorescence staining for γH2AX in liver sections from DMSO- or AAI-treated mice following treatment with either rSMOC2 or PBS.

**c** Representative immunofluorescence staining for cleaved caspase 3 in liver sections from DMSO- or AAI-treated mice following treatment with either rSMOC2 or PBS. n = 4 for the AAI + PBS and AAI + rSMOC2 groups.

**
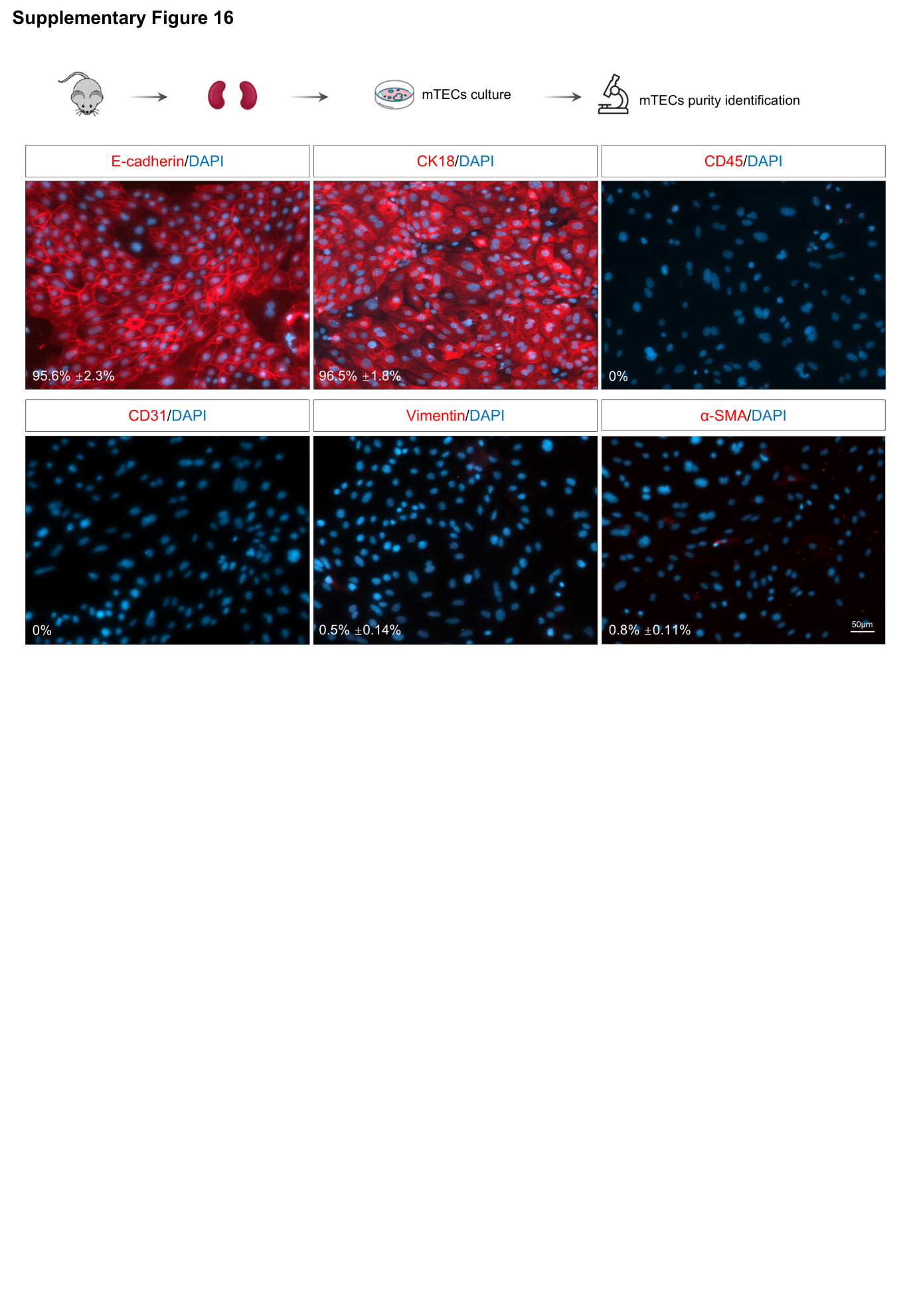
**

**Supplementary Fig. 16 Characterization of primary cultured mouse TECs using a panel of cell-type-specific markers.**

Primary TECs were freshly isolated from WT mice aged approximately 4-8 weeks and seeded in 12-well plates. Cells reached confluence within ~7 days and were subjected to immunofluorescence staining for the proximal tubular epithelial marker E-cadherin, the general epithelial marker CK18, the immune cell marker CD45, the mesenchymal cell markers Vimentin and α-SMA, and the endothelial marker CD31. Quantitative analysis revealed that more than 95% of cells were positive for E-cadherin or CK18, confirming high epithelial purity of the isolated mTECs.

**Supplementary tables**

**Table S1: Primers for PCR**

| **Gene** | **Forward (5’ to 3’)** | **Reverse (5’ to 3’)** |  |
| --- | --- | --- | --- |
| Smoc2 WT | TCTACACCCAAGCTTCCTTCATCCC | TTACACTGTCAGGAGACCATGAGCC | genotyping |
| Smoc2 KO | ACTTGCTTTAAAAAACCTCCCACA | ATCGGAAGACTGCTAAGTCCAAGGG | genotyping |
| mSmoc2 | GGAGCAGGGAAAGCAGATGAT | GAGTGTAGGGTAGCGTGAGG | qPCR |
| mGapdh | CGTGGAGTCTACTGGTGTCTTCA | GGCGGAGATGATGACCCTTT | qPCR |

**Table S2: Gene sets used for GSEA analysis**

| **Cell proliferation** | **Tubular injury** | **DNA repair** |
| --- | --- | --- |
| \| Ccnd1 \| Ccnk \| \| --- \| --- \| \| Ccnd2 \| Ccnj \| \| Mki67 \| Ccne2 \| \| Top2a \| Ccnc \| \| Plk1 \| Cnnm3 \| \| Ccnb1 \| Ccne1 \| \| Ccnt1 \| Ccnh \| \| Ccna2 \| Ccng2 \| \| Ccnd3 \| Ccno \| \| Ccnb2 \| Pcna \| \| Ccnl1 \| Cnnm4 \| \| Foxm1 \| Ccny \| \| Ccnt2 \| Ccnq \| \| Aurkb \| Cnnm1 \| \| Ccni \| Ccnl2 \| \| Cnnm2 \|  \| | \| Krt222 \| Krt7 \| \| --- \| --- \| \| Havcr1 \| Krt80 \| \| Vcam1 \| Akap12 \| \| Krt28 \| Lcn2 \| \| Il18 \| Krt23 \| \| Krt10 \| Atf3 \| \| Anxa3 \| Krt18 \| \| Igfbp7 \| Rbp4 \| \| Krt20 \| Krt15 \| \| Krt14 \| Krt8 \| \| Krt19 \| Timp2 \| \|  \|  \| \|  \| \|  \| \|  \| \|  \| | \| Fancd2os \|  \| Rpa3 \| Cetn2 \| Lig1 \| \| \| --- \| --- \| --- \| --- \| --- \| --- \| \| Cda \|  \| Gtf2h4 \| Gtf2h3 \| Ercc8 \| \| \| Xpa \|  \| Gtf2e2 \| Fancl \| Zfp182 \| \| \| Xrcc5 \|  \| Ddb1 \| Xpc \| Fanca \| \| \| Gtf2h2 \|  \| Cdk7 \| Rad51d \| Paxx \| \| \| Faap20 \|  \| Rad52 \| Brca1 \| Fance \| \| Xrcc2 \|  \| Xrcc1 \| Fancm \| Xab2 \| \| Mre11a \|  \| Uvssa \| Xrcc4 \| Fancb \| \| Nmnat1 \|  \| Rpa1 \| Rad23b \| Ehd4 \| \| Ccnh \|  \| Rad50 \| Rad51 \| Rpa2 \| \| \| \| Fan1 \|  \| Rad54l \| Rfc1 \| Zc3hc1 \| \| \| \| Gtf2h1 \|  \| Xrcc6 \| Fancc \| Rad51c \| \| \| \| Gtf2h5 \|  \| Parp1 \| Polh \| Ercc1 \| \| \| \| Palb2 \|  \| Birc5 \| Dyrk1a \| Faap100 \| \| \| Faap24 \|  \| Brca2 \| Ercc6 \| Dclre1c \| \| \| Xrcc3 \|  \| Fancg \| Mms19 \|  \| \| \|  \|  \|  \|  \| |

**Table S3: Primary antibodies for Western blot and immunofluorescence**

| **Primary Antibodies** | **Vendor** | **Catalog. No** | **Applications** | | |
| --- | --- | --- | --- | --- | --- |
|  |  |  | WB | IHC-P | ICC |
| γH2AX | Cell signaling | 9718 | 1:1000 | 1:250 |  |
| p-Rb Ser780 | Cell signaling | 8180 | 1:1000 |  |  |
| PARP | Cell signaling | 9542 | 1:1000 |  |  |
| Cleaved caspase3 | Cell signaling | 9661 | 1:1000 | 1:400 |  |
| CCND1 | Cell signaling | 55506 | 1:1000 | 1:200 |  |
| Ki-67 | Cell signaling | 9129 | 1:1000 | 1:250 |  |
| p-ERK1/2 | Cell signaling | 4370 | 1:2000 |  |  |
| ERK1/2 | Cell signaling | 4695 | 1:1000 |  |  |
| ITGB1 | Cell signaling | 9699 | 1:1000 |  |  |
| ITGB3 | Cell signaling | 13166 | 1:1000 |  | 1:100 |
| ITGB4 | Cell signaling | 14803 | 1:1000 |  |  |
| ITGB5 | Cell signaling | 3629 | 1:1000 |  |  |
| ITGA4 | Cell signaling | 8440 | 1:1000 |  |  |
| ITGA5 | Cell signaling | 4705 | 1:1000 |  |  |
| ITGAV | Cell signaling | 4711 | 1:1000 |  |  |
| Myc tag | Cell signaling | 2276 | 1:1000 |  | 1:400 |
| Villin1 | Cell signaling | 2369 |  | 1:150 |  |
| E-cadherin | Cell signaling | 3195 |  |  | 1:400 |
| CD31 | Cell signaling | 3528 |  |  | 1:300 |
| CD45 | BioLegend | 103111 |  |  | 1:50 |
| GAPDH | Abcam | Ab9485 | 1:2000 |  |  |
| Fibronectin | Abcam | Ab23750 | 1:3000 | 1:400 |  |
| Collagen I | Southern Biotech | 1310-01 | 1:500 |  |  |
| α-SMA | Sigma | A2547 | 1:5000 | 1:400 |  |
| Vimentin | Sigma | HPA001762 |  |  | 1:200 |
| SMOC2 | Home made | NA | 1:500 | 1:100 |  |
| SMOC2 | Affinity | DF14694 |  | 1:200 |  |
| Calbindin D28K | Proteintech | 66394 |  | 1:300 |  |
| Cytokeratin 18/CK18 | Proteintech | 10830-1-AP |  |  | 1:100 |
| AQP1 | Santa Cruz | 25287 |  | 1:100 |  |
| KIM1 | R&D | AF1817 |  | 1:250 |  |
| DBA (dye) | Vector Lab | FL-1031 |  | 1:50 |  |

**Table S4: Secondary antibodies for Western blot and immunofluorescence**

| **Secondary Antibodies** | **Vendor** | **Catalog. No** | **Applications** | | |
| --- | --- | --- | --- | --- | --- |
|  |  |  | WB | IHC-P | ICC |
| Anti-goat Alexa Fluor 488 | ThermoFisher | A-11055 |  | 1:300 |  |
| Anti-mouse Cy3 | Jackson Immunoresearch | 715-165-150 |  | 1:300 | 1:300 |
| Anti-rabbit Cy3 | Jackson Immunoresearch | 711-165-152 |  | 1:300 |  |
| Anti-rabbit Alexa Fluor 647 | Jackson Immunoresearch | 711-605-152 |  | 1:300 | 1:300 |
| Anti-mouse Alexa Fluor 647 | Jackson Immunoresearch | 115-605-003 |  | 1:300 |  |
| Anti-rabbit IgG-HRP | Santa Cruz | sc-2357 | 1:2000 |  |  |
| Anti-mouse IgG-HRP | Santa Cruz | sc-516102 | 1:2000 |  |  |
| Anti-goat IgG-HRP | Santa Cruz | sc-2354 | 1:2000 |  |  |

**References**

1. Pabla N, Gibson AA, Buege M, Ong SS, Li L, Hu S, Du G, Sprowl JA, Vasilyeva A, Janke LJ, Schlatter E, Chen T, Ciarimboli G, Sparreboom A. Mitigation of acute kidney injury by cell-cycle inhibitors that suppress both CDK4/6 and OCT2 functions. Proceedings of the National Academy of Sciences of the United States of America. 2015;112(16):5231-6. <https://doi.org/10.1073/pnas.1424313112>

2. Kim JY, Jayne LA, Bai Y, Feng M, Clark MA, Chung S, J WC, Cianciolo RE, Pabla NS. Ribociclib mitigates cisplatin-associated kidney injury through retinoblastoma-1 dependent mechanisms. Biochemical pharmacology. 2020;177:113939. <https://doi.org/10.1016/j.bcp.2020.113939>

3. Wen L, Wei Q, Livingston MJ, Dong G, Li S, Hu X, Li Y, Huo Y, Dong Z. PFKFB3 mediates tubular cell death in cisplatin nephrotoxicity by activating CDK4. Translational research : the journal of laboratory and clinical medicine. 2023;253:31-40. <https://doi.org/10.1016/j.trsl.2022.10.001>

4. Takaori K, Nakamura J, Yamamoto S, Nakata H, Sato Y, Takase M, Nameta M, Yamamoto T, Economides AN, Kohno K, Haga H, Sharma K, Yanagita M. Severity and Frequency of Proximal Tubule Injury Determines Renal Prognosis. Journal of the American Society of Nephrology : JASN. 2016;27(8):2393-406. <https://doi.org/10.1681/asn.2015060647>

5. Uphoff CC, Drexler HG. Detecting mycoplasma contamination in cell cultures by polymerase chain reaction. Methods in molecular biology (Clifton, NJ). 2011;731:93-103. <https://doi.org/10.1007/978-1-61779-080-5_8>
