# Supplementary material for: Matricellular protein SMOC2 safeguards tubular integrity in acute kidney injury via integrin β3-dependent inhibition of CCND1-CDK4/6 axis
Source: Mol Biomed. 2026 Feb 10;7:11. doi: 10.1186/s43556-026-00407-6 (PMC12886620; doi:10.1186/s43556-026-00407-6)

| **Sample File** | **Sample Name** | **Panel** | **Marker** | **Dye** | **Allele 1** | **Allele 2** | **Allele 3** | **Allele 4** |
| --- | --- | --- | --- | --- | --- | --- | --- | --- |
| HK2_Cell_authentification_Plate_001_2024-05-21.fsa | HK2 | GenePrint_10_v1.1 | AMEL | Y | X | Y |  | |
| HK2_Cell_authentification_Plate_001_2024-05-21.fsa | HK2 | GenePrint_10_v1.1 | CSF1PO | G | 13 |  |  | |
| HK2_Cell_authentification_Plate_001_2024-05-21.fsa | HK2 | GenePrint_10_v1.1 | D13S317 | G | 9 |  |  | |
| HK2_Cell_authentification_Plate_001_2024-05-21.fsa | HK2 | GenePrint_10_v1.1 | D16S539 | G | 12 |  |  | |
| HK2_Cell_authentification_Plate_001_2024-05-21.fsa | HK2 | GenePrint_10_v1.1 | D21S11 | B | 28 | 30 |  | |
| HK2_Cell_authentification_Plate_001_2024-05-21.fsa | HK2 | GenePrint_10_v1.1 | D5S818 | G | 12 |  |  | |
| HK2_Cell_authentification_Plate_001_2024-05-21.fsa | HK2 | GenePrint_10_v1.1 | D7S820 | G | 10 | 11 |  | |
| HK2_Cell_authentification_Plate_001_2024-05-21.fsa | HK2 | GenePrint_10_v1.1 | TH01 | B | 9 |  |  | |
| HK2_Cell_authentification_Plate_001_2024-05-21.fsa | HK2 | GenePrint_10_v1.1 | TPOX | Y | 8 | 9 |  | |
| HK2_Cell_authentification_Plate_001_2024-05-21.fsa | HK2 | GenePrint_10_v1.1 | vWA | Y | 17 | 18 |  | |
|  | | | | | | | | |
| **Sample File** | **Sample Name** | **Panel** | **Marker** | **Size 1** | **Size 2** | **Size 3** | **Size 4** | |
| HK2_Cell_authentification_Plate_001_2024-05-21.fsa | HK2 | GenePrint_10_v1.1 | AMEL | 104.05 | 109.75 |  |  | |
| HK2_Cell_authentification_Plate_001_2024-05-21.fsa | HK2 | GenePrint_10_v1.1 | CSF1PO | 345.68 |  |  |  | |
| HK2_Cell_authentification_Plate_001_2024-05-21.fsa | HK2 | GenePrint_10_v1.1 | D13S317 | 179.91 |  |  |  | |
| HK2_Cell_authentification_Plate_001_2024-05-21.fsa | HK2 | GenePrint_10_v1.1 | D16S539 | 290.82 |  |  |  | |
| HK2_Cell_authentification_Plate_001_2024-05-21.fsa | HK2 | GenePrint_10_v1.1 | D21S11 | 215.46 | 223.49 |  |  | |
| HK2_Cell_authentification_Plate_001_2024-05-21.fsa | HK2 | GenePrint_10_v1.1 | D5S818 | 134.77 |  |  |  | |
| HK2_Cell_authentification_Plate_001_2024-05-21.fsa | HK2 | GenePrint_10_v1.1 | D7S820 | 228.3 | 232.31 |  |  | |
| HK2_Cell_authentification_Plate_001_2024-05-21.fsa | HK2 | GenePrint_10_v1.1 | TH01 | 173.79 |  |  |  | |
| HK2_Cell_authentification_Plate_001_2024-05-21.fsa | HK2 | GenePrint_10_v1.1 | TPOX | 268.93 | 272.89 |  |  | |
| HK2_Cell_authentification_Plate_001_2024-05-21.fsa | HK2 | GenePrint_10_v1.1 | vWA | 150.24 | 154.21 |  |  | |

| **Sample File** | **Sample Name** | **Panel** | **Marker** | **Height 1** | **Height 2** | **Height 3 Height 4** |
| --- | --- | --- | --- | --- | --- | --- |
| HK2_Cell_authentification_Plate_001_2024-05-21.fsa | HK2 | GenePrint_10_v1.1 | AMEL | 4882 | 2639 | |
| HK2_Cell_authentification_Plate_001_2024-05-21.fsa | HK2 | GenePrint_10_v1.1 | CSF1PO | 15996 |  | |
| HK2_Cell_authentification_Plate_001_2024-05-21.fsa | HK2 | GenePrint_10_v1.1 | D13S317 | 7990 |  | |
| HK2_Cell_authentification_Plate_001_2024-05-21.fsa | HK2 | GenePrint_10_v1.1 | D16S539 | 14714 |  | |
| HK2_Cell_authentification_Plate_001_2024-05-21.fsa | HK2 | GenePrint_10_v1.1 | D21S11 | 10431 | 9329 | |
| HK2_Cell_authentification_Plate_001_2024-05-21.fsa | HK2 | GenePrint_10_v1.1 | D5S818 | 6712 |  | |
| HK2_Cell_authentification_Plate_001_2024-05-21.fsa | HK2 | GenePrint_10_v1.1 | D7S820 | 11613 | 5512 | |
| HK2_Cell_authentification_Plate_001_2024-05-21.fsa | HK2 | GenePrint_10_v1.1 | TH01 | 13958 |  | |
| HK2_Cell_authentification_Plate_001_2024-05-21.fsa | HK2 | GenePrint_10_v1.1 | TPOX | 4095 | 3881 | |
| HK2_Cell_authentification_Plate_001_2024-05-21.fsa | HK2 | GenePrint_10_v1.1 | vWA | 5840 | 9853 | |

**Cell line authentication** was performed by short tandem repeat (STR) profiling. Genomic DNA from HK-2 cells was analyzed using the GenePrint® 10 System, which examines nine autosomal STR loci (CSF1PO, D13S317, D16S539, D21S11, D5S818, D7S820, TH01, TPOX, and vWA) along with the amelogenin (AMEL) locus for sex determination. STR alleles were assigned based on fragment size (bp) and fluorescence peak height. The STR profile was subsequently queried against public reference databases using the STR Profile Search function available on the DSMZ CellDive platform (https://celldive.dsmz.de). This analysis revealed an 83.3% similarity to reference STR profiles, confirming a single-source human male STR profile and excluding cross-contamination or misidentification.


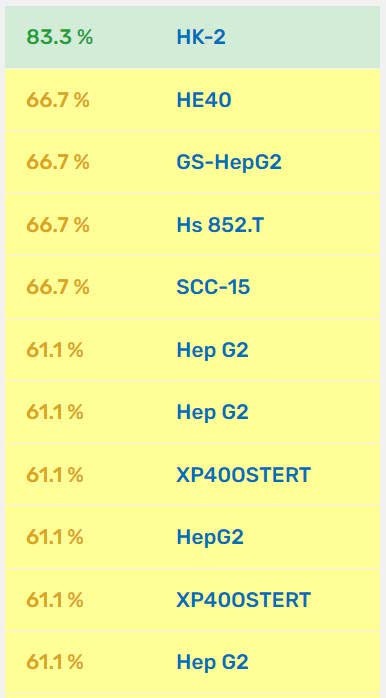

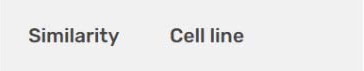

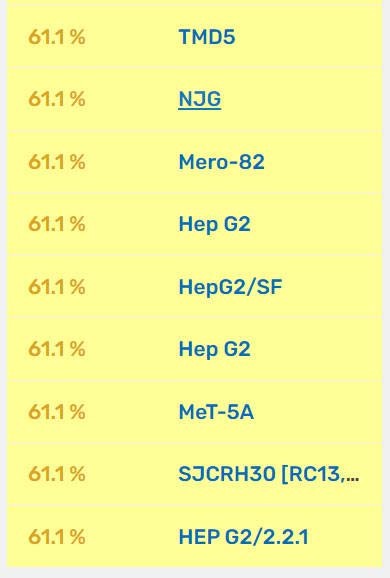

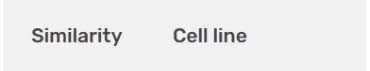


**Mycoplasma Detection by PCR**

Mycoplasma contamination was assessed by a PCR-based assay based on a previously published method (PMID: 21516400), targeting conserved regions of the mycoplasmal 16S rRNA gene using a set of broad-spectrum primers. Each PCR run included a plasmid-based positive control consisting of a pGEM plasmid containing the 16S rRNA PCR product of Acholeplasma laidlawii, a DNA preparation from mycoplasma-infected cell culture supernatant as an additional positive control, and a no-template negative control. PCR products were analyzed by agarose gel electrophoresis, and the presence of a band of the expected size was considered indicative of mycoplasma contamination. Randomly sampled culture supernatants from HK-2 cells tested negative for mycoplasma contamination.


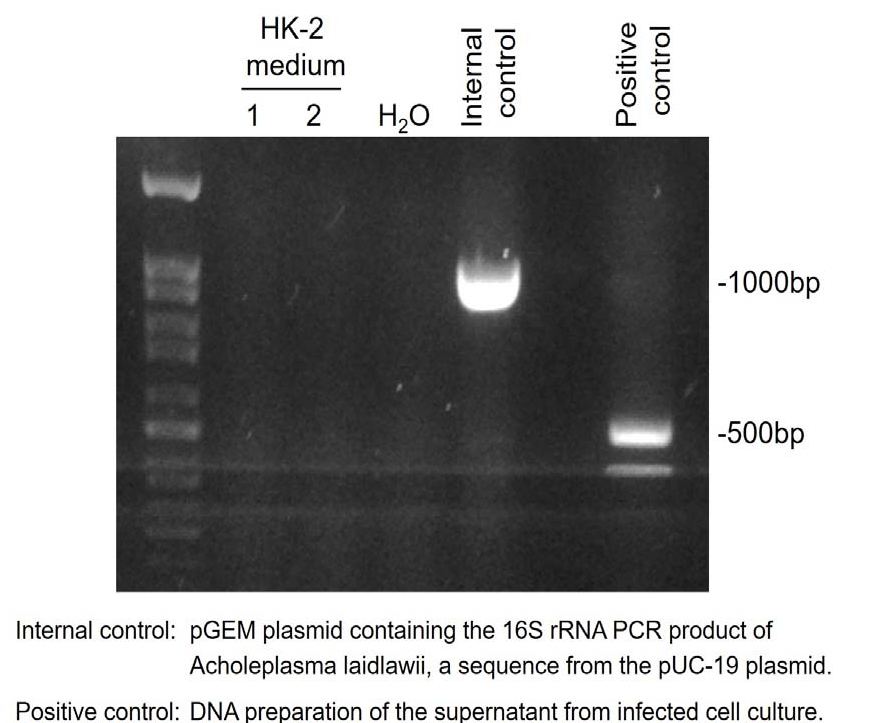

Supplement: Supplementary file 2 — Supplementary material 2. [file 43556_2026_407_MOESM2_ESM.docx]
